# Early Rhythm Versus Rate Control in Older Adults With New‐Onset Atrial Fibrillation: A Propensity Score–Matched Analysis

**DOI:** 10.1002/joa3.70400

**Published:** 2026-06-19

**Authors:** Faizan Ahmed, Najam Gohar, Hafsa Arshad Azam Raja, Haris Bin Tahir, Abdullah Masood, Anika Goel, Syed Mubeen Ahmed, Haziq Ahmed, Muhammad Imam, Ameer Haider Cheema, Ramsha Ali, Faseeh Haider, Fawaz Alenezi, Amro Taha

**Affiliations:** ^1^ Jersey Shore University Medical Centre Neptune New Jersey USA; ^2^ Ameer‐ud‐Din Medical College Lahore Pakistan; ^3^ Rawalpindi Medical University Rawalpindi Pakistan; ^4^ Lahore General Hospital Lahore Pakistan; ^5^ Kakatiya Medical College Warangal India; ^6^ Shaikh Khalifa Bin Zayed Al Nahyan Medical and Dental College Lahore Pakistan; ^7^ University of Texas Southwestern Medical Center Dallas Texas USA; ^8^ University of Illinois Chicago Illinois USA; ^9^ Allama Iqbal Medical College Lahore Pakistan; ^10^ Duke University School of Medicine Durham North Carolina USA; ^11^ West Virginia University Morgantown West Virginia USA

**Keywords:** atrial fibrillation, mortality, rate control, rhythm control, thromboembolism, ventricular arrhythmia

## Abstract

**Background:**

Atrial fibrillation (AF) is the most common sustained cardiac arrhythmia, associated with substantial morbidity and mortality. Although early rhythm control has demonstrated benefit in selected trial populations, its real‐world effectiveness remains uncertain.

**Methods:**

We conducted a retrospective multicenter cohort study using the TriNetX Global Collaborative Network. Adults (≥ 65 years) with newly diagnosed AF between 2005 and 2025 were included. Patients initiating rhythm control within 1 month of diagnosis were propensity score–matched 1:1 with rate control patients. The primary outcome was 1‐year all‐cause mortality. Secondary outcomes included thromboembolism, major bleeding, hospitalization, ventricular arrhythmia, cardiac arrest, syncope, AF recurrence, and cardioversion. Subgroup analyses evaluated modality‐specific rhythm control, contemporary era, and age‐stratified outcomes.

**Results:**

After matching, 200 631 patients were included in each group with balanced baseline characteristics. At 1 year, all‐cause mortality was higher with rhythm control than rate control (HR 1.20, 95% CI 1.18–1.22; *p* < 0.001). Rhythm control demonstrated higher rates of ventricular arrhythmia, cardiac arrest, hospitalization, syncope, and cardioversion, while thromboembolic events and major bleeding occurred less frequently. Substantial heterogeneity emerged by modality. Pharmacologic‐only subgroup revealed findings consistent with principal analysis, whereas procedural rhythm control showed lower mortality (HR 0.20, 95% CI 0.16–0.25) and hospitalization.

**Conclusions:**

Among older adults with newly diagnosed AF, early rhythm control was associated with higher mortality and arrhythmic complications versus rate control. Pharmacologic rhythm control largely drove excess mortality, whereas procedural approaches were linked to lower mortality and hospitalization. These findings underscore the importance of careful patient selection when considering early rhythm control strategies in older populations.

## Graphical abstract

In a large propensity score–matched real‐world cohort of older adults with newly diagnosed atrial fibrillation, early rhythm control was associated with lower thromboembolic and bleeding risk but higher mortality and arrhythmic complications compared with rate control. These findings highlight the importance of careful patient selection when considering rhythm‐control strategies in older populations.

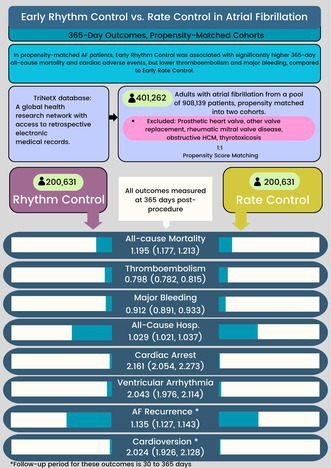

**Abbreviations**
AF: atrial fibrillation
CI: confidence interval
HCO: healthcare organization
HR: hazard ratio
PSM: propensity score matching
RD: risk difference
RR: risk ratio
SMD: standardized mean difference

## 1 Introduction

Atrial fibrillation (AF) is the most common sustained cardiac arrhythmia and affects more than 33 million people worldwide, markedly increasing the risk of stroke, heart failure, emergency department visits, and all‐cause mortality [1]. The treatment of AF primarily consists of two strategies: rate control, which targets the regulation of ventricular rate, and rhythm control, which seeks the restoration and maintenance of sinus rhythm with antiarrhythmic medication, cardioversion, or catheter ablation [2].

While randomized trials like AFFIRM found no significant mortality difference between the two strategies in earlier eras [3], recent studies, including the EAST‐AFNET 4 trial, indicate that early rhythm control can provide benefits to patients' cardiovascular health and safety in relation to stroke and/or cardiovascular death, particularly if done within 1 year of diagnosis of AF [4, 5]. However, these studies have looked at selected populations under trial conditions, raising concerns about external validity. Rhythm control has been perceived as underused for multiple reasons in routine practice, including proarrhythmic risk, drug toxicity, and limited long‐term benefit in elderly patients [6, 7]. Hence, there is some ambiguity regarding real‐world patient comparative effectiveness and safety of rhythm versus rate control strategy, especially early on post‐diagnosis of AF.

Observational studies conducted to date have often been limited in their sample size, patient population, and ability to adjust for confounding factors. Furthermore, several studies neglected to utilize an adequate matching method to account for baseline differences in comorbidities, disease severity, and treatment patterns. Even more concerning is that many studies lacked granular data to distinguish between hospitalizations for AF recurrence or downstream treatment dispositions that reflected true clinical practice [4, 8]. For these reasons, there is a need for larger population‐level studies that are reflective of clinical practice and apply statistical methods to control for residual confounding, such as propensity score–matching (PSM) methodology.

Thus, we report a retrospective cohort study utilizing the TriNetX global federated research platform, which extracts data from electronic health records of over 120 million patients across multiple healthcare organizations. Our approach takes advantage of the advances of real‐world data and PSM methodology to provide a meaningful comparison of 1‐year clinical outcomes, that is, all‐cause mortality, thromboembolism, bleeding, arrhythmias, and AF recurrence between patients who received early rhythm control and those who received early rate control.

## 2 Methods

### 2.1 Study Design and Data Source

We conducted a retrospective multicenter cohort study using the TriNetX global federated health research network (TriNetX LLC, Cambridge, MA, USA). This analysis utilized de‐identified electronic health record (EHR) data from the “Global Collaborative Network,” which initially included 161 and 160 participating healthcare organizations (HCOs) for the rhythm and rate control cohorts, respectively. The TriNetX platform aggregates patient‐level data across diagnoses, procedures, medications, and laboratory values, coded using standard terminologies (e.g., ICD‐10‐CM, CPT, RxNorm). All data are de‐identified in accordance with the Health Insurance Portability and Accountability Act (HIPAA) Safe Harbor provision (§164.514); thus, this study was exempt from Institutional Review Board approval. The analysis was performed and the dataset extracted on October 1, 2025. To ensure that every included patient had the opportunity for a complete 365‐day follow‐up window, eligibility for the index event was restricted to first qualifying AF diagnoses and medication initiation recorded on or before October 1, 2024; thus, patient recruitment for the cohort was closed at least 1 year prior to the analytic date. We followed the Strengthening the Reporting of Observational Studies in Epidemiology (STROBE) guidelines for reporting.

### 2.2 Cohort Definition

The study population consisted of adults aged ≥ 65 years with a new diagnosis of atrial fibrillation (AF). Two distinct cohorts were defined based on the initial treatment strategy within 1 month of the first instance of AF. The Early Rhythm Control cohort included patients whose first instance of rhythm control therapy occurred within 30 days after the first instance of AF. The Early Rate Control cohort included patients whose first instance of rate control therapy occurred within 30 days after the first instance of AF.

A 30‐day exposure window was selected to capture the initial therapeutic strategy following AF diagnosis and to better reflect the treating clinician's early management intent. In observational electronic health record datasets, longer exposure windows may introduce treatment crossover and exposure misclassification, as therapies initiated several months after diagnosis may represent treatment escalation or disease progression rather than the original management approach. Restricting the exposure window to 30 days also helps minimize immortal time bias, which can occur when patients must survive for extended periods before receiving the exposure of interest. Therefore, this window was chosen as a pragmatic approach to accurately distinguish early rhythm versus rate control strategies in real‐world clinical practice. The complete code list is provided in Table S1.

To ensure the specificity of the rate control cohort and minimize contamination from rhythm control strategies, an additional criterion was applied: patients in the rate control cohort must have had no evidence of rhythm control therapy within 1 year before or up to 1 year after their first AF diagnosis. The index date for each patient was defined as the date of the first qualifying AF diagnosis paired with the initiation of the respective control strategy (rhythm or rate) within the 30‐day exposure window, so that the index reflected both the new AF diagnosis and the start of rhythm or rate control therapy.

### 2.3 Inclusion Criteria

- Age ≥ 65 years.
- New‐onset atrial fibrillation was defined as the first ever recorded diagnosis of AF (ICD‐10‐CM codes I48.0, I48.1, I48.2, and I48.91).
- First instance of rhythm or rate control therapy occurring within 1 month after the first AF diagnosis.

### 2.4 Exclusion Criteria

Patients were excluded if they had any of the following conditions recorded before the index event, as they may contraindicate certain control strategies or confound outcomes: presence of a prosthetic heart valve (Z95.2) or other heart‐valve replacement (Z95.4), rheumatic mitral valve diseases (I05), obstructive hypertrophic cardiomyopathy (I42.1), and thyrotoxicosis (hyperthyroidism) (E05). The complete lists of codes for AF, rhythm control medications/procedures, and rate control medications are detailed in Table S1.

### 2.5 Outcomes

The primary and secondary outcomes were assessed from 1 day after the index event until 365 days post‐index to minimize immortal time bias. The prespecified outcomes were: all‐cause mortality, major bleeding, all‐cause hospitalization, ventricular arrhythmia, cardiac arrest, syncope, AF recurrence, and need for cardioversion. For transparency, per‐outcome settings regarding exclusion of patients with events before the time window are documented in Table S2, alongside detailed outcome definitions and codes used. Mortality was ascertained as all‐cause death only; cause‐of‐death information is not provided in a structured, harmonized format across contributing healthcare organizations in the TriNetX Global Collaborative Network, and cause‐specific mortality therefore could not be analyzed. Competing‐risk regression methods (e.g., Fine‐Gray subdistribution hazards) were not performed; the TriNetX Compare Outcomes analytic module reports Cox proportional hazards but does not include native competing‐risk estimators, and we therefore treated mortality as a single absorbing event in the time‐to‐event analyses. We discuss the implications of this approach for the interpretation of nonfatal outcomes in the Limitations.

### 2.6 Propensity Score Matching

To control for potential confounding due to nonrandom treatment assignment, we performed a 1:1 propensity score matching (PSM) without replacement using a caliper width of 0.1 times the pooled standard deviation of the logit of the propensity score. The propensity score was estimated using logistic regression, with cohort assignment as the dependent variable and a comprehensive set of baseline characteristics as covariates. Variables included in the PSM model were: *Demographics*: Age, sex, race, ethnicity; *Comorbidities*: Essential hypertension, heart failure, other cardiac arrhythmias, cardiomyopathy, diabetes mellitus, obesity etc.; *Procedures*: Critical care services; *Medications*: Beta‐blockers, antiarrhythmics, ACE inhibitors, antianginals, calcium channel blockers, angiotensin II receptor blockers, diuretics, anticoagulants, and platelet aggregation inhibitors; and *Laboratory Values*. Balance between cohorts before and after matching was assessed using standardized mean differences (SMD), with an SMD < 0.10 considered indicative of good balance. Full covariate code lists appear in Table S3.

### 2.7 Statistical Analysis

All analyses were performed within the TriNetX platform. Baseline characteristics were summarized as means with standard deviations for continuous variables and counts with percentages for categorical variables. Pre‐ and post‐matching comparisons used *t*‐tests for continuous variables and chi‐square tests for categorical variables. For outcomes, we conducted three primary analyses. (1) Risk analysis: we calculated the risk, risk difference, and risk ratio with 95% confidence intervals (CIs) for each outcome in the matched cohorts. (2) Survival analysis: Kaplan–Meier survival curves were constructed for all time‐to‐event outcomes, with between‐cohort comparisons performed using the log‐rank test, and hazard ratios (HRs) with 95% CIs were derived from Cox proportional hazards regression. (3) Number‐of‐instances analysis: the mean number of outcome events per patient was compared between cohorts using *t*‐tests. A two‐sided *p* value < 0.05 was considered statistically significant for all hypothesis tests. The TriNetX platform automatically accounts for censoring at the last known activity date within a patient's record.

### 2.8 Subgroup and Sensitivity Analyses

To address the heterogeneity inherent in real‐world rhythm and rate control strategies and the evolving landscape of atrial fibrillation management, three prespecified secondary analyses were performed in addition to the principal comparison.

#### 2.8.1 Modality‐Specific Rhythm Control Subgroups

The Early Rhythm Control cohort was redefined as two mutually exclusive modality‐specific subgroups, using the medication and procedure codes listed in Table S1. The Pharmacologic‐Only subgroup included patients who initiated a Class IC or Class III antiarrhythmic drug within 30 days of the first AF diagnosis and who had no catheter ablation procedure within the same window. The Procedural subgroup included patients who underwent catheter ablation within 30 days of the first AF diagnosis. Each subgroup was independently propensity score matched 1:1 to the same Early Rate Control comparator using the identical covariate set, caliper width, and balance criteria described above. The full set of prespecified outcomes was reevaluated in each subgroup using risk analysis, Kaplan–Meier survival analysis, and Cox proportional hazards regression as described above.

#### 2.8.2 Contemporary‐Era Sensitivity Analysis

To examine whether the principal findings were robust to changes in atrial fibrillation management over the recruitment window, a sensitivity analysis was performed restricted to patients whose index AF diagnosis occurred between January 1, 2015 and October 1, 2024. This contemporary period was selected because it reflects a coherent and clinically meaningful era of AF care: by the end of 2015, all four direct oral anticoagulants (dabigatran, rivaroxaban, apixaban, and edoxaban) had received United States Food and Drug Administration approval and had largely supplanted warfarin as the predominant stroke‐prevention strategy; the 2014 AHA/ACC/HRS guideline for the management of patients with AF had formally positioned rhythm control alongside rate control and provided explicit guidance on antiarrhythmic drug selection in patients with structural heart disease and renal impairment; cryoballoon ablation, which had received regulatory approval in late 2010, achieved parity with radiofrequency ablation in clinical use following the FIRE AND ICE trial in 2016; and dronedarone prescribing had been reshaped by the 2011 PALLAS trial and subsequent FDA labeling restrictions in patients with permanent AF and advanced heart failure. Within this contemporary subcohort, the full propensity score–matching procedure was repeated using the same covariate set and matching specifications described above, and the primary and secondary outcomes were reestimated to assess whether the direction and magnitude of the principal findings were preserved in a more contemporary practice setting.

#### 2.8.3 Age‐Stratified Analyses

To evaluate effect modification across the heterogeneous older‐adult population, prespecified age‐stratified analyses were performed in three clinically conventional strata: young‐old (65–74 years), old‐old (75–84 years), and oldest‐old (85 years and older). Within each stratum, the Early Rhythm Control and Early Rate Control cohorts were independently propensity score matched 1:1 using the same covariate set and matching specifications applied to the overall analysis, and the outcomes were reestimated using the analytic approaches described above.

For all subgroup and sensitivity analyses, covariate balance was reassessed after matching using standardized mean differences, with SMD < 0.10 considered indicative of adequate balance.

## 3 Results

### 3.1 Study Population

**FIGURE 1:**
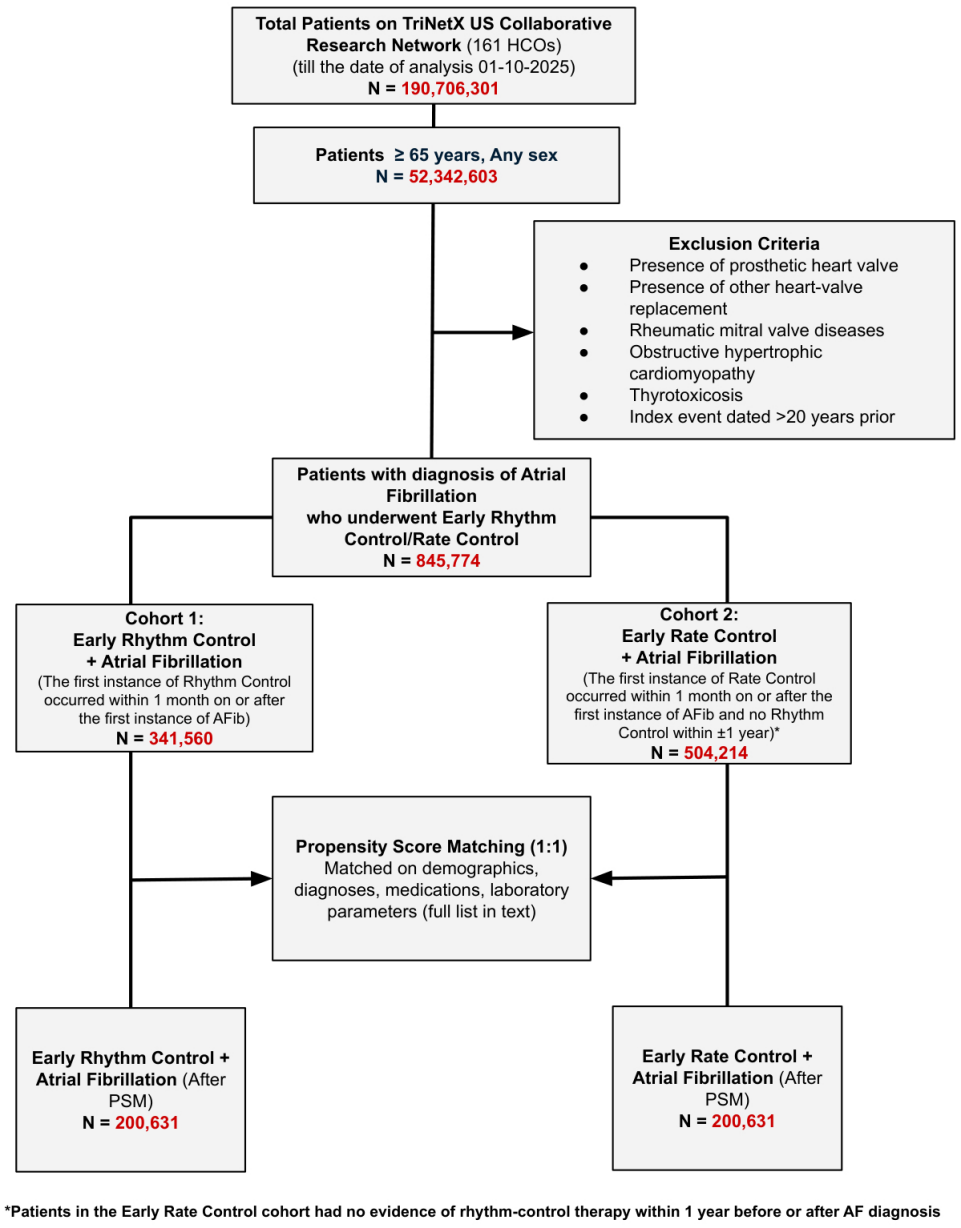
Flowchart of patient selection process. *Patients in the Early Rate Control cohort had no evidence of rhythm‐control therapy with 1 year before or after AF diagnosis.

We identified 908 139 patients meeting initial criteria from inception up to 20 years before October 1, 2025. After applying exclusions for prosthetic heart valve, other valve replacement, rheumatic mitral valve disease, obstructive hypertrophic cardiomyopathy, and thyrotoxicosis, 341 560 subjects were assigned to the Early Rhythm Control cohort and 504 214 to the Early Rate Control cohort. The index date was defined as the first qualifying rhythm or rate control event with a minimum follow‐up of 365 days after the index event. The selection process is shown in Figure 1.

### 3.2 Patient Characteristics

In the unmatched cohort, Early Rhythm Control patients were younger (mean 73.2 ± 8.9 vs. 75.8 ± 9.3 years; SMD 0.288) and had higher prevalences of obesity (17.3% vs. 9.8%; SMD 0.22), other cardiac arrhythmias (20.0% vs. 10.4%; SMD 0.27), heart failure (28.9% vs. 14.8%), and chronic ischemic heart disease (35.1% vs. 16.3%) compared with Early Rate Control.

After 1:1 propensity score matching, baseline covariates were well balanced (SMDs < 0.10) for age (73.8 ± 9.1 vs. 74.0 ± 9.3), male sex (53.8% vs. 53.9%), overweight/obesity (11.1% vs. 10.8%), and other arrhythmias (12.8% vs. 12.3%). Cohort sizes were 200 631 in each cohort after matching. Baseline characteristics are summarized in Table 1 (see Table S4 for detailed baseline characteristics).

**TABLE 1:** Selected baseline characteristics of the study cohorts before and after propensity score matching.

	Before propensity matching				After propensity matching			
	Early rhythm control (*n* = 341 560)	Early rate control (*n* = 504 214)	*p*	SMD	Early rhythm control (*n* = 200 631)	Early rate control (*n* = 200 631)	*p*	SMD
**Demographics**								
Age	73.2 ± 8.9	75.8 ± 9.3	< 0.001	0.29	73.8 ± 9.1	74.0 ± 9.3	< 0.001	0.02
White	248 344 (72.7%)	368 096 (73.0%)	0.003	0.007	144 975 (72.3%)	145 258 (72.4%)	0.318	0.003
Male	189 807 (55.6%)	256 849 (50.9%)	< 0.001	0.09	107 911 (53.8%)	108 106 (53.9%)	0.537	0.002
**Diagnosis**								
Hypertension	178 492 (52.3%)	199 243 (39.5%)	< 0.001	0.26	78 932 (39.3%)	78 770 (39.3%)	0.601	0.002
Heart failure	98 829 (28.9%)	74 858 (14.8%)	< 0.001	0.35	35 631 (17.8%)	34 570 (17.2%)	< 0.001	0.01
Other cardiac arrhythmias	68 287 (20.0%)	52 517 (10.4%)	< 0.001	0.27	25 621 (12.8%)	24 768 (12.3%)	< 0.001	0.01
Cardiomyopathy	29 494 (8.6%)	14 891 (3.0%)	< 0.001	0.24	8497 (4.2%)	8260 (4.1%)	0.061	0.006
Diabetes mellitus	90 860 (26.6%)	90 710 (18.0%)	< 0.001	0.21	37 935 (18.9%)	37 577 (18.7%)	0.148	0.005
Cerebral infarction	24 370 (7.1%)	34 418 (6.8%)	< 0.001	0.01	13 647 (6.8%)	13 902 (6.9%)	0.111	0.005
Ischemic heart disease	119 880 (35.1%)	82 393 (16.3%)	< 0.001	0.44	40 467 (20.2%)	39 989 (19.9%)	0.059	0.006
Atherosclerosis	28 366 (8.3%)	19 339 (3.8%)	< 0.001	0.19	9105 (4.5%)	8996 (4.5%)	0.407	0.003
Chronic kidney disease	68 137 (19.9%)	58 427 (11.6%)	< 0.001	0.23	26 488 (13.2%)	25 921 (12.9%)	0.008	0.008
Acute myocardial infarction	49 109 (14.4%)	24 145 (4.8%)	< 0.001	0.33	13 397 (6.7%)	13 282 (6.6%)	0.466	0.002
Dyslipidemias	154 424 (45.2%)	154 025 (30.5%)	< 0.001	0.31	64 210 (32.0%)	63 425 (31.6%)	0.008	0.008
Presence of aortocoronary bypass graft	25 104 (7.3%)	12 931 (2.6%)	< 0.001	0.22	6555 (3.3%)	6572 (3.3%)	0.880	< 0.001
Presence of cardiac pacemaker	10 303 (3.0%)	13 552 (2.7%)	< 0.001	0.02	5581 (2.8%)	5574 (2.8%)	0.946	< 0.001
**Medications**								
Beta‐blockers/related	175 428 (51.4%)	40 143 (8.0%)	< 0.001	1.08	36 966 (18.4%)	38 136 (19.0%)	< 0.001	0.015
Antiarrhythmics	138 620 (40.6%)	108 735 (21.6%)	< 0.001	0.42	52 154 (26.0%)	51 244 (25.5%)	0.001	0.01
Calcium channel blockers	118 787 (34.8%)	53 009 (10.5%)	< 0.001	0.61	33 434 (16.7%)	32 535 (16.2%)	< 0.001	0.01
Anticoagulants	189 154 (55.4%)	131 884 (26.2%)	< 0.001	0.62	68 047 (33.9%)	65 746 (32.8%)	< 0.001	0.02
Platelet aggregation inhibitors	142 522 (41.7%)	89 128 (17.7%)	< 0.001	0.55	44 876 (22.4%)	44 546 (22.2%)	0.211	0.004
**Laboratory**								
Heart rate	82.4 ± 22.4	79.8 ± 17.9	< 0.001	0.13	81.2 ± 21.8	80.0 ± 18.0	< 0.001	0.06
Systolic blood pressure	120.6 ± 26.6	129.3 ± 23.1	< 0.001	0.35	123.3 ± 25.3	128.7 ± 23.6	< 0.001	0.22
Diastolic blood pressure	66.6 ± 16.2	71.0 ± 14.3	< 0.001	0.28	68.0 ± 15.5	70.5 ± 14.6	< 0.001	0.17
Troponin I	1.9 ± 12.4	0.8 ± 9.6	< 0.001	0.10	1.4 ± 11.0	1.0 ± 9.9	< 0.001	0.04
BNP	1012.2 ± 3430.1	814.2 ± 2814.4	< 0.001	0.06	871.1 ± 3131.6	903.5 ± 3084.2	0.246	0.01
INR	1.3 ± 0.6	1.4 ± 0.8	< 0.001	0.10	1.3 ± 0.7	1.3 ± 0.7	0.174	0.007

Abbreviations: BNP, brain natriuretic peptide; INR, international standardized ratio; SMD, standardized mean difference.

### 3.3 Primary Outcome

**FIGURE 2:**
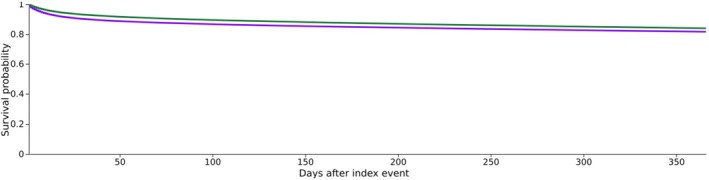
Kaplan–Meier survival analysis plot for all‐cause mortality (Purple = Early Rhythm Control, Green = Early Rate Control).

At 365 days, the pooled Early Rhythm Control cohort demonstrated a higher observed risk of all‐cause mortality compared with the Early Rate Control cohort. All‐cause mortality occurred in 15.7% of patients receiving Early Rhythm Control versus 13.2% of patients receiving Early Rate Control, with RR 1.19 (95% CI 1.18–1.21), RD 0.03, *p* < 0.001. Kaplan–Meier survival probability at 365 days was 81.64% for Early Rhythm Control versus 83.98% for Early Rate Control, with a between‐cohort log‐rank *p* < 0.001 and a Cox‐derived hazard ratio of 1.20 (95% CI 1.18–1.22). The Kaplan–Meier curve is shown in Figure 2.

However, substantial heterogeneity emerged according to rhythm‐control modality in subgroup analyses. Pharmacologic rhythm‐control strategies were associated with higher mortality (HR 1.25), whereas procedural rhythm‐control strategies demonstrated substantially lower mortality (HR 0.20) relative to rate control. These findings suggest that the pooled rhythm‐control analysis may obscure clinically important differences between pharmacologic and procedural rhythm‐control approaches (Table 2).

**TABLE 2:** Comparison of outcomes after propensity score matching.

Outcome	Atrial fibrillation (AF)		RD (95% CI)	RR (95% CI)	HR (95% CI)	*p*
	Early rhythm control	Early rate control				
All‐cause mortality	31 587/200631	26 433/200631	0.03 (0.02 to 0.03)	1.19 (1.18 to 1.21)	1.20 (1.18 to 1.22)	< 0.001
Thromboembolism	14 452/200631	18 104/200631	−0.02 (−0.02 to −0.02)	0.80 (0.78 to 0.81)	0.79 (0.77 to 0.81)	< 0.001
Major bleeding	12 516/200631	13 728/200631	−0.006 (−0.008 to −0.005)	0.91 (0.89 to 0.93)	0.91 (0.89 to 0.93)	< 0.001
All‐cause hospitalizations	79 972/200631	77 721/200631	0.01 (0.008 to 0.01)	1.03 (1.02 to 1.04)	1.04 (1.03 to 1.05)	< 0.001
Cardiac arrest	4680/200631	2166/200631	0.01 (0.01 to 0.01)	2.16 (2.05 to 2.27)	2.18 (2.07 to 2.29)	< 0.001
Ventricular arrhythmia	9929/200631	4859/200631	0.02 (0.02 to 0.03)	2.04 (1.98 to 2.11)	2.08 (2.01 to 2.15)	< 0.001
Syncope	8300/200631	7784/200631	0.003 (0.001 to 0.004)	1.07 (1.03 to 1.10)	1.07 (1.04 to 1.11)	< 0.001
AF recurrences^a^	93 523/209460	82 430/209460	0.05 (0.05 to 0.06)	1.13 (1.13 to 1.14)	1.23 (1.22 to 1.24)	< 0.001
Cardioversion^a^	4569/209460	2257/209460	0.01 (0.01 to 0.01)	2.02 (1.93 to 2.13)	2.06 (1.96 to 2.16)	< 0.001

Abbreviations: CI, confidence interval; HR, hazard ratio; RD, risk difference; RR, risk ratio.

^a^ Follow‐up period for these outcomes is 30–365 days.

### 3.4 Secondary Outcomes

#### 3.4.1 Thromboembolism

Thromboembolism occurred in 14 452 of 200 631 (7.2%) Early Rhythm Control patients versus 18 104 of 200 631 (9.0%) Early Rate Control patients (RR 0.80, 95% CI 0.78–0.81; RD −0.02, 95% CI −0.02 to −0.02; *p* < 0.001; HR 0.79, 95% CI 0.77–0.81; log‐rank *p* < 0.001).

#### 3.4.2 Major Bleeding

Among all at‐risk patients, a major bleeding event occurred in 12 516 of 200 631 (6.2%) Early Rhythm Control patients versus 13 728 of 200 631 (6.8%) Early Rate Control patients (RR 0.91, 95% CI 0.89–0.93; RD −0.006, 95% CI −0.008 to −0.005; *p* < 0.001; HR 0.91, 95% CI 0.89–0.93; log‐rank *p* < 0.001).

#### 3.4.3 All‐Cause Hospitalization

All‐cause hospitalization occurred in 79 972 of 200 631 (39.9%) Early Rhythm Control patients versus 77 721 of 200 631 (38.7%) Early Rate Control patients (RR 1.03, 95% CI 1.02–1.04; RD 0.01, 95% CI 0.008–0.01; *p* < 0.001; HR 1.04, 95% CI 1.03–1.05; log‐rank *p* < 0.001).

#### 3.4.4 Cardiac Arrest

Cardiac arrest occurred in 4680 of 200 631 (2.3%) Early Rhythm Control patients compared with 2166 of 200 631 (1.1%) Early Rate Control patients (RR 2.16, 95% CI 2.05–2.27; RD 0.01, 95% CI 0.01–0.01; *p* < 0.001; HR 2.18, 95% CI 2.07–2.29; log‐rank *p* < 0.001).

#### 3.4.5 Ventricular Arrhythmia

This outcome occurred in 9929 of 200 631 (5.0%) Early Rhythm Control patients versus 4859 of 200 631 (2.4%) Early Rate Control patients (RR 2.04, 95% CI 1.98–2.11; RD 0.02, 95% CI 0.02–0.03; *p* < 0.001; HR 2.08, 95% CI 2.01–2.15; log‐rank *p* < 0.001).

#### 3.4.6 Syncope

Among at‐risk patients, syncope occurred in 8300 of 200 631 (4.1%) Early Rhythm Control patients versus 7784 of 200 631 (3.9%) Early Rate Control patients (RR 1.07, 95% CI 1.03–1.10; RD 0.003, 95% CI 0.001–0.004; *p* < 0.001; HR 1.07, 95% CI 1.04–1.11; log‐rank *p* < 0.001).

#### 3.4.7 AF Recurrence

AF recurred in 93 523 of 209 460 (44.6%) Early Rhythm Control patients versus 82 430 of 209 460 (39.4%) Early Rate Control patients (RR 1.13, 95% CI 1.13–1.14; RD 0.05, 95% CI 0.05–0.06; *p* < 0.001; HR 1.23, 95% CI 1.22–1.24; log‐rank *p* < 0.001).

#### 3.4.8 Cardioversion

**FIGURE 3:**
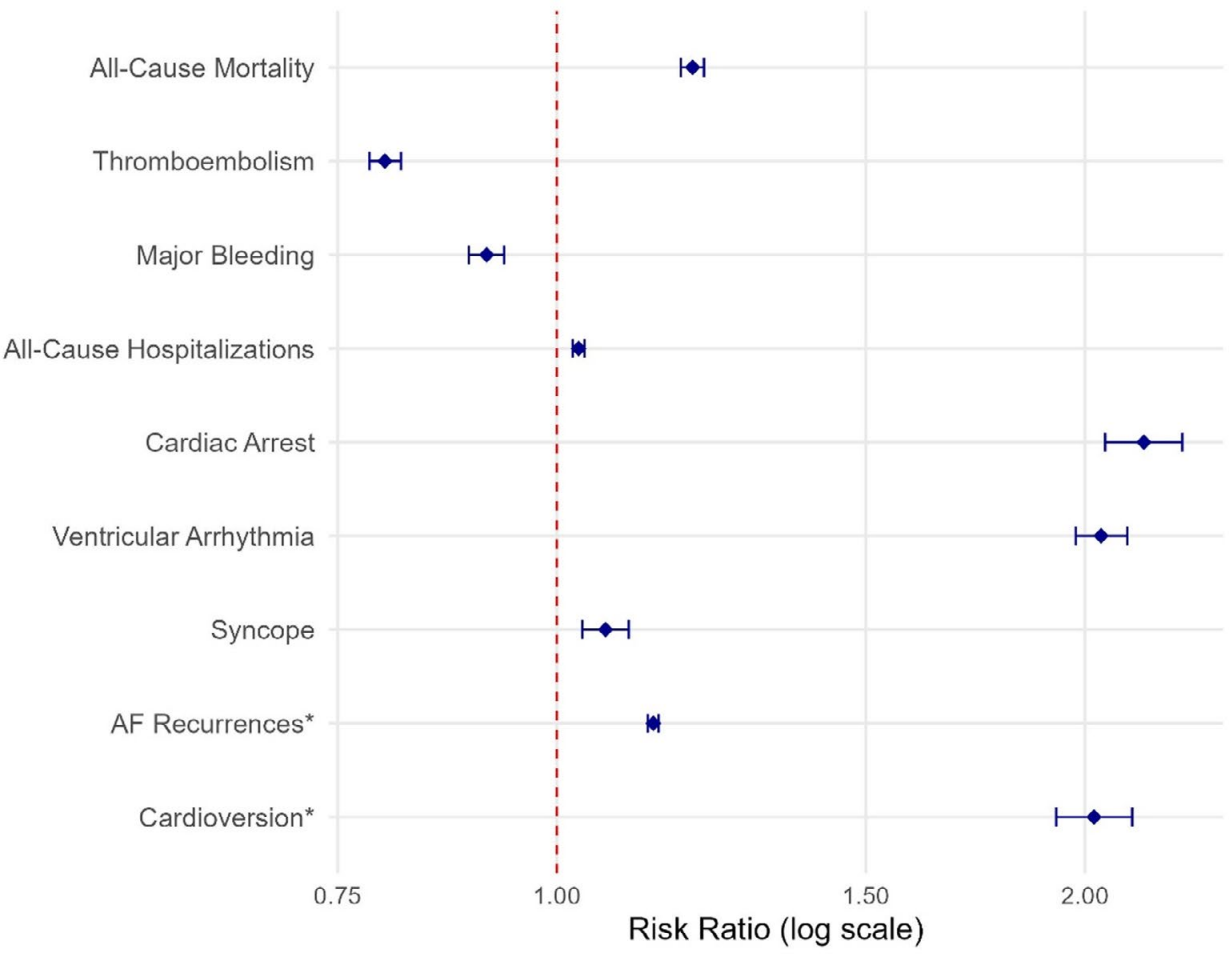
Forest plot illustrating the relative risks and 95% confidence intervals for the evaluated outcomes in early rhythm versus early rate control strategies. AF, atrial fibrillation. *Follow‐up period for these outcomes is 30–365 days.

**FIGURE 4:**
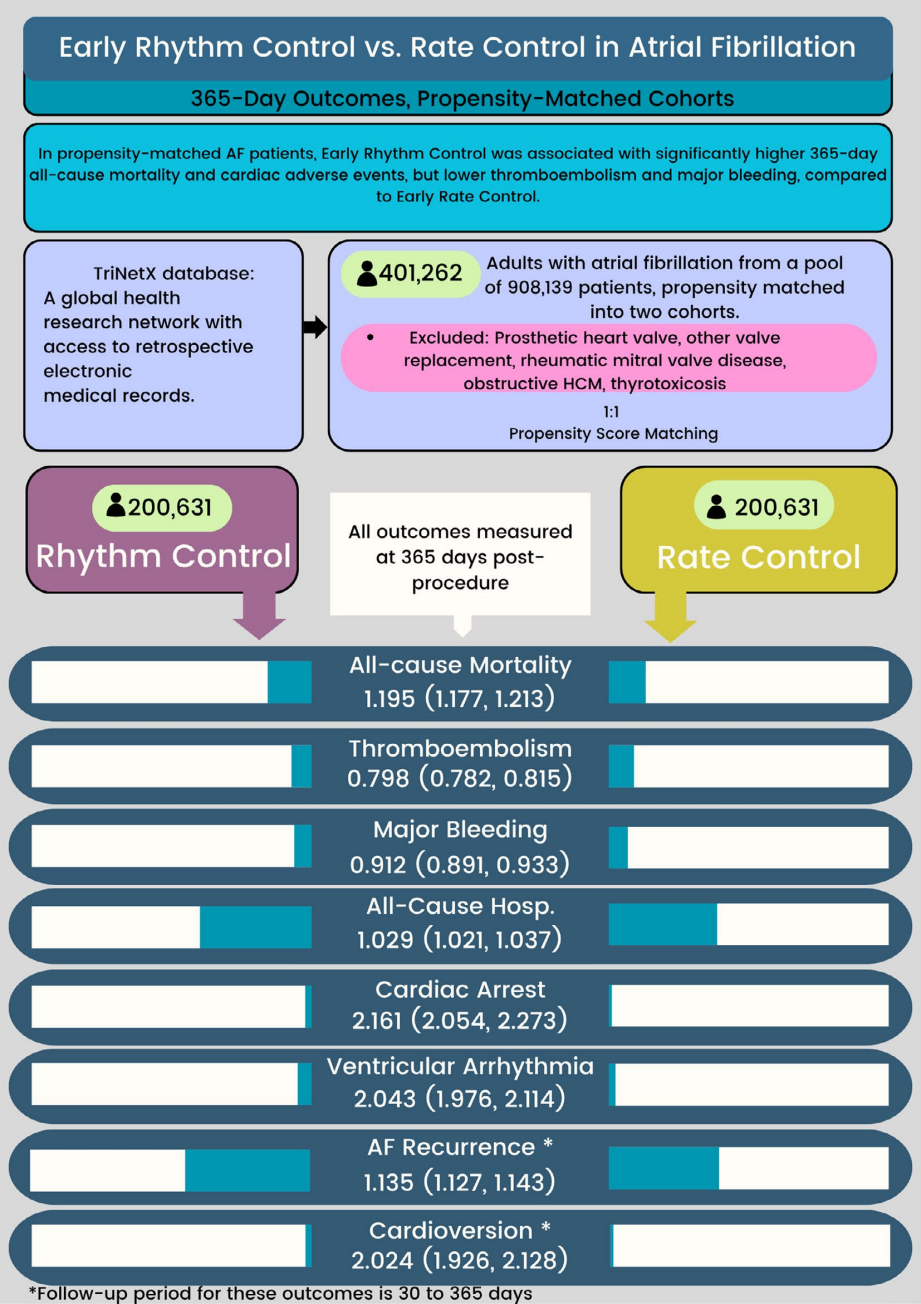
Graphical abstract summarizing the key outcomes of early rhythm control versus early rate control in patients with atrial fibrillation. *Follow‐up period for these outcomes is 30–365 days.

Cardioversion occurred in 4569 of 209 460 (2.2%) Early Rhythm Control patients versus 2257 of 209 460 (1.1%) Early Rate Control patients (RR 2.02, 95% CI 1.93–2.13; RD 0.01, 95% CI 0.01–0.01; *p* < 0.001; HR 2.06, 95% CI 1.96–2.16; log‐rank *p* < 0.001). The forest plot of relative risks for the evaluated outcomes is presented in Figure 3. The Kaplan–Meier survival analysis plots for secondary outcomes are shown in Figure S1. A graphical abstract summarizing the key findings of the study is presented in Figure 4.

### 3.5 Modality‐Specific Subgroup Analyses

In the Pharmacologic‐only Early Rhythm Control subgroup (181 591 matched patients per arm), the direction and magnitude of every primary and secondary outcome were consistent with the principal analysis, with all‐cause mortality (HR 1.25, 95% CI 1.23–1.27), all‐cause hospitalization (HR 1.03), ventricular arrhythmia (HR 2.13), and cardiac arrest (HR 2.32) remaining elevated and thromboembolism (HR 0.82) and major bleeding (HR 0.94) remaining lower in the rhythm arm.

In the Procedural Early Rhythm Control subgroup (5986 matched patients per arm), the pattern of effect for the most clinically important outcomes diverged sharply from the principal analysis: all‐cause mortality was substantially lower with procedural rhythm control (HR 0.20, 95% CI 0.16–0.25), as were thromboembolism (HR 0.40), all‐cause hospitalization (HR 0.60), and cardiac arrest (HR 0.68, not statistically significant), while ventricular arrhythmia (HR 1.33) remained modestly elevated and AF recurrence (HR 1.37) and cardioversion (HR 2.63) were higher, reflecting the procedural pathway. The full set of outcome estimates after propensity score matching for both modality subgroups is provided in Tables S5 and S6.

These findings demonstrate substantial heterogeneity across rhythm‐control modalities. Pharmacologic rhythm‐control strategies appeared to account for much of the excess mortality, hospitalization, cardiac arrest, and ventricular arrhythmia observed in the pooled rhythm‐control cohort. In contrast, procedural rhythm‐control strategies demonstrated more favorable mortality and hospitalization associations with comparatively attenuated arrhythmic risk estimates. These results underscore that rhythm control is not a uniform therapeutic strategy, and pooled comparisons with rate control may mask clinically important differences across modalities.

### 3.6 Contemporary‐Era Sensitivity Analysis (2015–2024)

After restricting the cohort to patients with an index AF diagnosis between January 1, 2015 and October 1, 2024 and re‐performing 1:1 propensity score matching (154 932 matched patients per arm), the principal findings were preserved in both direction and magnitude across every outcome, with all‐cause mortality remaining higher with rhythm control (HR 1.27, 95% CI 1.25–1.29) and the differential pattern for thromboembolism, ventricular arrhythmia, and cardiac arrest essentially unchanged. The persistence of effect direction and magnitude in the post‐DOAC, contemporary‐era cohort supports the consistency of the principal findings to era heterogeneity. Outcome estimates are presented in Table S7.

### 3.7 Age‐Stratified Analyses

Across all three prespecified age strata, the direction of effect was preserved relative to the principal analysis, but the absolute magnitude of the mortality association narrowed with advancing chronologic age: the HR for all‐cause mortality with rhythm control was 1.45 (95% CI 1.40–1.50) in the young‐old (65–74 years; 52 223 matched per arm), 1.26 (95% CI 1.23–1.30) in the old‐old (75–84 years; 67 146 matched per arm), and 1.14 (95% CI 1.12–1.17) in the oldest‐old (85+ years; 63 240 matched per arm). The relative excesses of ventricular arrhythmia and cardiac arrest in the rhythm arm and the relative reduction in thromboembolism in the rhythm arm were qualitatively preserved across all three age strata. Outcome estimates for each age stratum are provided in Tables S8–S10.

## 4 Discussion

In this large propensity‐matched cohort of nearly 400 000 atrial fibrillation patients using the TriNetX Global Collaborative Network, Early Rhythm Control was associated with higher 1‐year all‐cause mortality, increased risks of ventricular arrhythmia and cardiac arrest compared with Early Rate Control. However, it significantly reduced thromboembolic events and major bleeding compared to Early Rate Control. AF recurrences and cardioversion procedures were notably more frequent with Rhythm Control, indicating greater rhythm instability. Syncope and hospitalizations were slightly higher, suggesting increased healthcare utilization. Importantly, subgroup analyses demonstrated substantial heterogeneity according to rhythm‐control modality, with pharmacologic rhythm‐control strategies largely mirroring the overall findings, whereas procedural rhythm control exhibited a markedly different outcome profile, including lower mortality and hospitalization. These findings underscore that rhythm control should not be interpreted as a uniform therapeutic strategy and highlight a trade‐off between thromboembolic protection and arrhythmic safety in rhythm‐based strategies.

### 4.1 Mortality and Ventricular Arrhythmias

The most striking finding was the significantly higher 1‐year all‐cause mortality in the “Early Rhythm Control” group (15.7% vs. 13.2%; RR 1.19), despite balanced baseline characteristics. This finding diverges from prior meta‐analyses, including that conducted by Siddiqi et al. [9], which have generally reported neutral or favorable mortality outcomes with rhythm control strategies and lower mortality as reported by Al‐Sadawi et al. [10] and Han et al. [11]. Similarly, the AFFIRM trial, CASTLE‐AF, and RAFT‐AF trial have reported comparable effects of rhythm versus rate control in terms of mortality [12, 13, 14, 15]. The elevated mortality observed here may reflect real‐world complexities such as older age, comorbidity burden, procedural risks, and rhythm instability that are often underrepresented in controlled trial settings [16, 17]. Kaplan–Meier survival analysis demonstrated early and persistent separation of survival curves, with a 365‐day survival probability of 81.6% in the rhythm control group compared with 84.0% in the rate control group, reinforcing the robustness of the hazard ratio (HR 1.20, 95% CI 1.18–1.22) and underscores that excess risk emerges soon after therapy initiation and persists throughout follow‐up.

The higher mortality observed with early rhythm control in our study contrasts with findings from randomized trials such as EAST‐AFNET 4, which demonstrated improved cardiovascular outcomes with early rhythm control strategies. The observational design of this study precludes causal inference, and the mortality association should be interpreted as hypothesis‐generating rather than evidence that rhythm‐control therapy itself increases mortality. Several factors may explain this discrepancy. First, our study population consisted exclusively of adults aged ≥ 65 years with a high burden of comorbidities, whereas trial populations were younger and more carefully selected. Cause‐specific mortality was unavailable, and non‐cardiovascular deaths may have contributed to the observed association [17]. Older patients with structural heart disease, heart failure, or renal impairment may be more susceptible to the proarrhythmic and systemic adverse effects of antiarrhythmic medications, which could contribute to increased mortality in routine clinical practice [16]. Second, treatment strategies in real‐world settings are heterogeneous and may include variable adherence, differences in medication selection, and inconsistent monitoring compared with the standardized protocols used in clinical trials [2, 7]. Third, residual confounding and treatment selection bias may persist despite propensity score matching; clinicians may preferentially pursue rhythm control in patients with more symptomatic or advanced disease, potentially enriching the rhythm cohort with higher‐risk individuals [4]. Finally, procedural complications related to rhythm restoration strategies, including cardioversion or catheter‐based interventions, may also contribute to the observed increase in arrhythmic events and mortality in an older, comorbid population [5]. Together, these factors highlight the challenges of translating trial‐based rhythm control strategies into real‐world clinical practice and underscore the importance of careful patient selection when considering early rhythm control in older adults. Competing‐risk analyses were not performed, further limiting interpretation. These differences highlight that our findings should not be considered definitive but rather hypothesis‐generating, reflecting the complexities and potential biases inherent to real‐world observational data.

Subgroup analyses revealed important heterogeneity. Pharmacologic‐only rhythm control mirrored the principal analysis, with consistently higher mortality (HR 1.25), ventricular arrhythmia (HR 2.13), and cardiac arrest (HR 2.32), alongside reduced thromboembolism (HR 0.82) and bleeding (HR 0.94). In contrast, procedural rhythm control (catheter ablation) diverged sharply, showing substantially lower mortality (HR 0.20), thromboembolism (HR 0.40), and hospitalization (HR 0.60), though AF recurrence (HR 1.37) and cardioversion (HR 2.63) were higher. These results suggest that procedural approaches may mitigate mortality risk while still carrying rhythm instability burdens, whereas pharmacologic strategies appear to account for much of the mortality association observed in the overall rhythm‐control cohort. However, the markedly lower mortality observed in the procedural subgroup should be interpreted with caution, as it may partly reflect treatment‐selection bias and residual confounding. Patients selected for catheter ablation may differ from those managed with rate control in ways that are incompletely captured within electronic health record data despite propensity score matching. This divergence underscores that rhythm control is not a unified strategy, and outcomes differ substantially depending on modality. Therefore, the overall findings should be interpreted in the context of this heterogeneity rather than as the effect of a single rhythm‐control strategy.

Consistent with this interpretation, the increased mortality risk may also be partially explained by the markedly higher incidence of ventricular arrhythmia (RR 2.04) and cardiac arrest (RR 2.16) in the “Early Rhythm Control” group. These arrhythmic safety signals suggest that rhythm restoration efforts, whether pharmacologic or procedural, may introduce proarrhythmic risks either due to different classes of antiarrhythmic drugs or cardiac catheterization, particularly in vulnerable populations such as reported by different studies [18, 19, 20]. Additionally, the higher AF recurrence rate and cardioversion burden point to rhythm instability and treatment failure, which may contribute to adverse outcomes through repeated hospitalizations, medication adjustments, and procedural complications, which are in line with results reported by AlTurki et al. [21]. The higher incidence of syncope may further reflect transient rhythm instability, medication‐related hypotension, or bradyarrhythmia episodes [22]. These observations highlight the importance of careful arrhythmic surveillance and individualized risk stratification when initiating rhythm‐control strategies.

### 4.2 Consistent Benefits in Thromboembolic and Bleeding Outcomes

Our study aligns with prior evidence in demonstrating reduced thromboembolic events and major bleeding with rhythm control, as reported by Wong et al. [23], where anticoagulation therapy is used post‐cardioversion in patients with atrial fibrillation and flutter. These benefits likely stem from improved atrial mechanical function, reduced stasis, and more stable anticoagulation regimens [24]. The exclusion of patients with prior events strengthens the validity of these incident outcome estimates and supports the mechanistic rationale for rhythm restoration.

Despite the elevated mortality, Rhythm Control demonstrated favorable effects on thromboembolism and major bleeding, consistent with improved atrial mechanical function and anticoagulation stability. However, it is in contrast with the meta‐analyses conducted by Zafeiropoulos et al. [25] and Romiti et al. [26] that demonstrated a reduced mortality: unlike our result and a reduced risk of stroke and thromboembolic events. These benefits highlight the therapeutic potential of rhythm control but underscore the importance of patient selection. In younger or lower‐risk populations, the thromboembolic advantages may outweigh arrhythmic risks, whereas in older adults with structural heart disease, rate control may offer a safer mortality profile.

### 4.3 AF Recurrence, Cardioversion Burden, and Utilization

Our study uniquely quantified AF recurrence and cardioversion burden, revealing substantial rhythm instability and procedural demand in the rhythm control cohort. AF recurrence was notably higher with Rhythm Control, accompanied by a twofold increase in cardioversion procedures. The results are similar to the AFFIRM trial [26]. In contrast to our study, the EAST‐AFNET trial reports that an early rhythm control strategy reduced composite cardiovascular endpoints with a more preserved sinus rhythm [27]. Additionally, the CASTLE‐AF trial demonstrated a sustained reduction in AF burden [28]. These findings underscore the challenges of maintaining a durable sinus rhythm in older adults and suggest that rhythm control may not achieve sustained efficacy in routine practice. The higher AF recurrence rate observed in the rhythm‐control group is difficult to interpret. Patients undergoing rhythm‐control strategies generally receive more intensive rhythm monitoring and follow‐up, including device‐based surveillance, which increases detection of recurrent AF episodes compared with rate‐control patients. Therefore, recurrence outcomes may not accurately reflect biological recurrence risk but rather differences in monitoring intensity between groups. The procedural burden and repeated interventions may contribute to increased healthcare utilization and patient distress as reported by Greene et al. [29] However, early intervention may reduce hospitalization costs [30]. All‐cause hospitalizations were slightly higher in the Rhythm Control group, potentially driven by procedural follow‐up, arrhythmic complications, and recurrence management [31, 32, 33]. While the absolute difference was modest, it may have implications for resource allocation and care coordination, especially in older populations with limited physiological reserve.

Secondary outcomes also showed consistent divergence in Kaplan–Meier curves. Thromboembolism (HR 0.79) and major bleeding (HR 0.91) were significantly reduced in the rhythm control group, whereas ventricular arrhythmia (HR 2.08), cardiac arrest (HR 2.18), AF recurrence (HR 1.23), cardioversion (HR 2.06), syncope (HR 1.07), and hospitalization (HR 1.04) were all more frequent. The survival plots for these outcomes demonstrated that excess arrhythmic and hospitalization risks emerged early and persisted throughout follow‐up, while thromboembolic and bleeding protection remained durable. Additionally, age‐stratified analyses demonstrated attenuation of the mortality excess with advancing age: HR 1.45 in the young‐old (65–74 years), HR 1.26 in the old‐old (75–84 years), and HR 1.14 in the oldest‐old (≥ 85 years). Kaplan–Meier curves across strata showed consistent relative increases in arrhythmic events and reductions in thromboembolism, but the absolute mortality gap narrowed with age, suggesting that comorbidity burden and frailty may dilute the impact of rhythm control strategies in the oldest patients.

### 4.4 Strengths and Limitations

This study offers several strengths that enhance its credibility and clinical relevance. Leveraging the TriNetX Global Collaborative Network, it draws from a large, diverse, multicenter real‐world population of over 400 000 older adults with newly diagnosed atrial fibrillation, enabling robust comparative effectiveness analysis. Methodological rigor was ensured through prespecified cohort definitions, standardized follow‐up windows, and comprehensive 1:1 propensity score matching, which achieved strong covariate balance and minimized confounding. Excluding patients with prior outcomes strengthened internal validity by focusing on incident events. The inclusion of Kaplan–Meier survival analyses provided temporal insight into outcome divergence, demonstrating that excess mortality and arrhythmic risk with rhythm control emerged early and persisted throughout follow‐up. Furthermore, extensive subgroup and sensitivity analyses including pharmacologic versus procedural rhythm control, contemporary DOAC era restriction, and age‐stratified cohorts enhanced the robustness and external validity of the findings, highlighting heterogeneity in treatment effects across modalities and patient subgroups. The study captured a wide range of clinically meaningful endpoints, including mortality, thromboembolism, bleeding, arrhythmias, and healthcare utilization, offering a holistic view of treatment impact. These design features, along with a transparent analytic framework, support reproducibility and nuanced interpretation of rhythm versus rate control strategies in routine care.

Nonetheless, several limitations warrant consideration. Despite robust matching, residual confounding and confounding by indication remain important sources of bias. In routine clinical practice, patients selected for rhythm‐control strategies may differ systematically from those managed with rate control in ways that are not fully captured within electronic health record data. Factors such as AF subtype, symptom burden, left atrial size, left ventricular ejection fraction, frailty, functional status, socioeconomic factors, and physician treatment preference may influence both treatment selection and subsequent outcomes. Consequently, the observed mortality differences may partially reflect underlying differences in patient risk profiles rather than a direct effect of the treatment strategy itself. Although most baseline characteristics achieved adequate balance, minor residual imbalance remained for certain variables, including systolic and diastolic blood pressure (SMD > 0.10), which may reflect differences in underlying cardiovascular risk profiles between treatment groups. No additional post‐matching regression adjustment analyses were performed; therefore, the potential influence of these residual imbalances on the observed associations cannot be completely excluded. Modality‐specific subgroup analyses were performed; however, the TriNetX platform does not provide granular procedural detail regarding specific ablation techniques, antiarrhythmic drug dosing, operator experience, longitudinal treatment crossover, or adherence patterns, which may influence clinical outcomes. Outcomes were defined using diagnostic codes, which may vary across institutions; however, excluding patients with prior events helps mitigate misclassification. Additionally, AF recurrence may be influenced by surveillance bias, as patients undergoing rhythm‐control strategies may receive more intensive monitoring, increasing the likelihood of detecting recurrent arrhythmias. AF recurrences and cardioversion were assessed from day 30 to 365, potentially missing early events. TriNetX also censors patients at their last known activity date, which may result in incomplete outcome capture. Additionally, cause‐specific mortality data were unavailable within TriNetX, and non‐cardiovascular deaths may have contributed to the observed mortality associations in this older adult population. Finally, the study population was limited to patients aged ≥ 65 years, which may restrict generalizability to younger cohorts or nonnetwork settings.

## 5 Conclusion

In this large, propensity score–matched, real‐world cohort of older adults with newly diagnosed atrial fibrillation, early rhythm control was associated with a lower incidence of thromboembolic events and major bleeding but a higher risk of 1‐year all‐cause mortality and serious arrhythmic complications compared with rate control. Kaplan–Meier survival curves confirmed early and sustained divergence in outcomes, while subgroup analyses revealed that the observed mortality association was predominantly seen among patients receiving pharmacologic rhythm‐control strategies, whereas procedural approaches such as catheter ablation were associated with lower mortality and thromboembolic risk. Sensitivity analyses in the contemporary DOAC era and age‐stratified cohorts demonstrated consistency of these associations, with attenuation of mortality excess in the oldest patients. Given the observational nature of this study and the potential for residual confounding and treatment‐selection bias, these findings should be interpreted as hypothesis‐generating rather than causal. Treatment decisions should therefore be individualized, with careful consideration of patient comorbidities and close monitoring when rhythm‐control strategies are selected. Further studies are needed to better define which patient subgroups may derive net benefit from early rhythm control in real‐world settings.

## Author Contributions

Faizan Ahmed contributed to conceptualization, methodology, and supervision of the study and reviewed and edited the manuscript. Najam Gohar contributed to conceptualization, methodology, statistical analysis, formal analysis, and drafted the original manuscript. Hafsa Arshad Azam Raja contributed to data curation, validation, and manuscript review and editing. Haris Bin Tahir contributed to validation and manuscript review and editing. Abdullah Masood contributed to statistical analysis, data curation, and manuscript review and editing. Anika Goel and Syed Mubeen Ahmed contributed to investigation, data interpretation, and manuscript review and editing. Haziq Ahmed contributed to data curation, visualization, and manuscript review and editing. Muhammad Imam contributed to data curation, validation, and manuscript review and editing. Ameer Haider Cheema contributed to formal analysis, validation, and manuscript review and editing. Ramsha Ali contributed to investigation, data interpretation, and manuscript review and editing. Faseeh Haider contributed to supervision, methodology, and manuscript review and editing. Fawaz Alenezi contributed to supervision and project administration and reviewed the manuscript. Amro Taha contributed to supervision, project administration, and manuscript review and editing. All authors read and approved the final manuscript.

## Funding

The authors have nothing to report.

## Disclosure

No previously published material, figures, or tables requiring permission for reproduction were included in this manuscript.

## Ethics Statement

This study utilized de‐identified data from the TriNetX Research Network, a federated network of electronic health record databases. All data are de‐identified in compliance with the Health Insurance Portability and Accountability Act (HIPAA) Privacy Rule. As the study involved only de‐identified data and no direct patient interaction, institutional review board approval and informed consent were not required.

## Consent

Patient consent was not required because the study used only fully de‐identified data obtained from the TriNetX database.

## Conflicts of Interest

The authors declare no conflicts of interest.

## Supporting information


**Figure S1:** Kaplan–Meier survival analysis plots for 1‐year outcomes.
**Table S1:** Participants eligibility with the relevant used codes.
**Table S2:** Outcome definition and codes used for identification.
**Table S3:** Baseline variables with their relevant codes.
**Table S4:** Detailed baseline characteristics of the study cohort before and after propensity score matching.
**Table S5:** Comparison of outcomes after propensity score matching for subgroup Pharmacological Stratified.
**Table S6:** Comparison of outcomes after propensity score matching for subgroup Procedure Stratified.
**Table S7:** Comparison of outcomes after propensity score matching for subgroup 2015–2024 Stratified.
**Table S8:** Comparison of outcomes after propensity score matching for subgroup Age 65–74 years Stratified.
**Table S9:** Comparison of outcomes after propensity score matching for subgroup Age 75–84 years Stratified.
**Table S10:** Comparison of outcomes after propensity score matching for subgroup Age 85 years or above Stratified.

## Data Availability

The data that support the findings of this study are available from the TriNetX Research Network. Due to licensing restrictions and data use agreements, raw data are not publicly available. Access to the TriNetX platform can be obtained through institutional subscription. Aggregate data supporting the findings of this study may be available from the corresponding author upon reasonable request and with permission from TriNetX.
