# Supplementary material for: Early Rhythm Versus Rate Control in Older Adults With New‐Onset Atrial Fibrillation: A Propensity Score–Matched Analysis
Source: J Arrhythm. 2026 Jun 19;42(3):e70400. doi: 10.1002/joa3.70400 (PMC13282267; doi:10.1002/joa3.70400)
Supplement: Supplementary file 1 — Figure S1: Kaplan–Meier survival analysis plots for 1‐year outcomes. Table S1: Participants eligibility with the relevant used codes. Table S2: Outcome definition and codes used for identification. Table S3: Baseline variables with their relevant codes. Table S4: Detailed baseline characteristics of the study cohort before and after propensity score matching. Table S5: Comparison of outcomes after propensity score matching for subgroup Pharmacological Stratified. Table S6: Comparison of outcomes after propensity score matching for subgroup Procedure Stratified. Table S7: Comparison of outcomes after propensity score matching for subgroup 2015–2024 Stratified. Table S8: Comparison of outcomes after propensity score matching for subgroup Age 65–74 years Stratified. Table S9: Comparison of outcomes after propensity score matching for subgroup Age 75–84 years Stratified. Table S10: Comparison of outcomes after propensity score matching for subgroup Age 85 years or above Stratified. [file JOA3-42-e70400-s001.docx]

**Supplementary Material**

**Table S1:** Participants eligibility with the relevant used codes

**Table S2:** Outcome definition and codes used for identification

**Table S3:** Baseline variables with their relevant codes

**Table S4:** Detailed baseline characteristics of the study cohort before and after propensity score matching

**Table S5:** Comparison of outcomes after propensity score matching for subgroup Pharmacological Stratified

**Table S6:** Comparison of outcomes after propensity score matching for subgroup Procedure Stratified

**Table S7:** Comparison of outcomes after propensity score matching for subgroup 2015-2024 Stratified

**Table S8:** Comparison of outcomes after propensity score matching for subgroup Age 65-74 years Stratified

**Table S9:** Comparison of outcomes after propensity score matching for subgroup Age 75-84 years Stratified

**Table S10:** Comparison of outcomes after propensity score matching for subgroup Age 85 years or above Stratified

**Figure S1:** Kaplan-Meier Survival Analysis Plots for 1-Year Outcomes

**Table S1: Participants eligibility with the relevant used codes**

Cohort 1: Early Rhythm Control + Atrial Fibrillation

Cohort 2: Early Rate Control + Atrial Fibrillation

**Cohort 1: Early Rhythm Control + Atrial Fibrillation**

|  | | | | | |
| --- | --- | --- | --- | --- | --- |
| Ungrouped terms | | | | | |
|  | must have |  | demographics | Age | Age (at least 65 years (most recent occurrence)) |
| Group 1 | | | | | |
|  | **Group 1A AFib** | | | | |
|  | must have | any of | diagnosis | UMLS:ICD10CM:I48.91 | Unspecified atrial fibrillation |
|  |  |  | diagnosis | UMLS:ICD10CM:I48.1 | Persistent atrial fibrillation |
|  |  |  | diagnosis | UMLS:ICD10CM:I48.2 | Chronic atrial fibrillation |
|  |  |  | diagnosis | UMLS:ICD10CM:I48.0 | Paroxysmal atrial fibrillation |
|  | date constraint | | This group occurred before 1 year ago | | |
|  | event relationship | | The first instance of Rhythm Control occurred within 1 month on or after the first instance of AFib | | |
|  | **Group 1B Rhythm Control** | | | | |
|  | must have | any of | medication | NLM:RXNORM:4441 | flecainide |
|  |  |  | medication | NLM:RXNORM:49247 | dofetilide |
|  |  |  | medication | NLM:RXNORM:703 | amiodarone |
|  |  |  | medication | NLM:RXNORM:8754 | propafenone |
|  |  |  | medication | NLM:RXNORM:9947 | sotalol |
|  |  |  | medication | NLM:RXNORM:233698 | dronedarone |
|  |  |  | procedure | UMLS:CPT:93657 | Additional linear or focal intracardiac catheter ablation of the left or right atrium for treatment of atrial fibrillation remaining after completion of pulmonary vein isolation (List separately in addition to code for primary procedure) |
|  |  |  | procedure | UMLS:CPT:93656 | Comprehensive electrophysiologic evaluation including transseptal catheterizations, insertion and repositioning of multiple electrode catheters with intracardiac catheter ablation of atrial fibrillation by pulmonary vein isolation, including intracardiac electrophysiologic 3-dimensional mapping, intracardiac echocardiography including imaging supervision and interpretation, induction or attempted induction of an arrhythmia including left or right atrial pacing/recording, right ventricular pacing/recording, and His bundle recording, when performed |
| Group 2 | | | | | |
|  | **Exclusions** | | | | |
|  | cannot have |  | diagnosis | UMLS:ICD10CM:Z95.2 | Presence of prosthetic heart valve |
|  |  | or | diagnosis | UMLS:ICD10CM:Z95.4 | Presence of other heart-valve replacement |
|  |  | or | diagnosis | UMLS:ICD10CM:I05 | Rheumatic mitral valve diseases |
|  |  | or | diagnosis | UMLS:ICD10CM:I42.1 | Obstructive hypertrophic cardiomyopathy |
|  |  | or | diagnosis | UMLS:ICD10CM:E05 | Thyrotoxicosis [hyperthyroidism] |
|  | date constraint | | This group occurred before 1 year ago | | |

**Cohort 2: Early Rate Control + Atrial Fibrillation**

| Ungrouped terms | | | | | |
| --- | --- | --- | --- | --- | --- |
|  | must have |  | demographics | Age | Age (at least 65 years (most recent occurrence)) |
| Group 1 | | | | | |
|  | **Group 1A AFib** | | | | |
|  | must have | any of | diagnosis | UMLS:ICD10CM:I48.91 | Unspecified atrial fibrillation |
|  |  |  | diagnosis | UMLS:ICD10CM:I48.1 | Persistent atrial fibrillation |
|  |  |  | diagnosis | UMLS:ICD10CM:I48.2 | Chronic atrial fibrillation |
|  |  |  | diagnosis | UMLS:ICD10CM:I48.0 | Paroxysmal atrial fibrillation |
|  | date constraint | | This group occurred before 1 year ago | | |
|  | event relationship | | The first instance of Rate Control occurred within 1 month on or after the first instance of AFib | | |
|  | **Group 1B Rate Control** | | | | |
|  | must have | any of | medication | NLM:RXNORM:1202 | atenolol |
|  |  |  | medication | NLM:RXNORM:19484 | bisoprolol |
|  |  |  | medication | NLM:RXNORM:20352 | carvedilol |
|  |  |  | medication | NLM:RXNORM:49737 | esmolol |
|  |  |  | medication | NLM:RXNORM:6918 | metoprolol |
|  |  |  | medication | NLM:RXNORM:8787 | propranolol |
|  |  |  | medication | NLM:RXNORM:11170 | verapamil |
|  |  |  | medication | NLM:RXNORM:3443 | diltiazem |
|  |  |  | medication | NLM:RXNORM:3407 | digoxin |
| Group 2 | | | | | |
|  | **Group 2A AFib** | | | | |
|  | must have | any of | diagnosis | UMLS:ICD10CM:I48.0 | Paroxysmal atrial fibrillation |
|  |  |  | diagnosis | UMLS:ICD10CM:I48.1 | Persistent atrial fibrillation |
|  |  |  | diagnosis | UMLS:ICD10CM:I48.2 | Chronic atrial fibrillation |
|  |  |  | diagnosis | UMLS:ICD10CM:I48.91 | Unspecified atrial fibrillation |
|  | date constraint | | This group occurred before 1 year ago | | |
|  | event relationship | | The first instance of No Rhythm Control occurred within 1 year before or up to 1 year after the first instance of AFib | | |
|  | **Group 2B No Rhythm Control** | | | | |
|  | cannot have |  | medication | NLM:RXNORM:4441 | flecainide |
|  |  | or | medication | NLM:RXNORM:49247 | dofetilide |
|  |  | or | medication | NLM:RXNORM:703 | amiodarone |
|  |  | or | procedure | UMLS:CPT:93657 | Additional linear or focal intracardiac catheter ablation of the left or right atrium for treatment of atrial fibrillation remaining after completion of pulmonary vein isolation (List separately in addition to code for primary procedure) |
|  |  | or | medication | NLM:RXNORM:8754 | propafenone |
|  |  | or | medication | NLM:RXNORM:9947 | sotalol |
|  |  | or | medication | NLM:RXNORM:233698 | dronedarone |
|  |  | or | procedure | UMLS:CPT:93656 | Comprehensive electrophysiologic evaluation including transseptal catheterizations, insertion and repositioning of multiple electrode catheters with intracardiac catheter ablation of atrial fibrillation by pulmonary vein isolation, including intracardiac electrophysiologic 3-dimensional mapping, intracardiac echocardiography including imaging supervision and interpretation, induction or attempted induction of an arrhythmia including left or right atrial pacing/recording, right ventricular pacing/recording, and His bundle recording, when performed |
| Group 3 | | | | | |
|  | **Exclusions** | | | | |
|  | cannot have |  | diagnosis | UMLS:ICD10CM:Z95.2 | Presence of prosthetic heart valve |
|  |  | or | diagnosis | UMLS:ICD10CM:Z95.4 | Presence of other heart-valve replacement |
|  |  | or | diagnosis | UMLS:ICD10CM:I05 | Rheumatic mitral valve diseases |
|  |  | or | diagnosis | UMLS:ICD10CM:I42.1 | Obstructive hypertrophic cardiomyopathy |
|  |  | or | diagnosis | UMLS:ICD10CM:E05 | Thyrotoxicosis [hyperthyroidism] |
|  | date constraint | | This group occurred before 1 year ago | | |

**Table S2: Outcome definition and codes used for identification**

| All-Cause Mortality | | | | |
| --- | --- | --- | --- | --- |
|  | **Outcome definition** | | | |
|  | | Demographics | Deceased | Deceased |
|  | **Settings for the performed analyses** | | | |
|  | | Risk analysis | | including patients with outcome prior to the time window |
|  | | Kaplan - Meier survival analysis | | including patients with outcome prior to the time window |
|  | | Number of instances analysis | | including patients with outcome prior to the time window including patients with zero outcomes counts are grouped by date |
| Thromboembolism | | | | |
|  | **Outcome definition** | | | |
|  | | Diagnosis | UMLS:ICD10CM:I63 | Cerebral infarction |
|  | | Diagnosis | UMLS:ICD10CM:G45 | Transient cerebral ischemic attacks and related syndromes |
|  | | Diagnosis | UMLS:ICD10CM:I74 | Arterial embolism and thrombosis |
|  | **Settings for the performed analyses** | | | |
|  | | Risk analysis | | including patients with outcome prior to the time window |
|  | | Kaplan - Meier survival analysis | | including patients with outcome prior to the time window |
|  | | Number of instances analysis | | including patients with outcome prior to the time window including patients with zero outcomes counts are grouped by date |
| Major Bleeding (Composite) | | | | |
|  | **Outcome definition** | | | |
|  | | Diagnosis | UMLS:ICD10CM:I60 | Nontraumatic subarachnoid hemorrhage |
|  | | Diagnosis | UMLS:ICD10CM:I61 | Nontraumatic intracerebral hemorrhage |
|  | | Diagnosis | UMLS:ICD10CM:I62 | Other and unspecified nontraumatic intracranial hemorrhage |
|  | | Diagnosis | UMLS:ICD10CM:K92.0 | Hematemesis |
|  | | Diagnosis | UMLS:ICD10CM:K92.1 | Melena |
|  | | Diagnosis | UMLS:ICD10CM:K92.2 | Gastrointestinal hemorrhage, unspecified |
|  | | Diagnosis | UMLS:ICD9CM:578 | Gastrointestinal hemorrhage |
|  | **Settings for the performed analyses** | | | |
|  | | Risk analysis | | including patients with outcome prior to the time window |
|  | | Kaplan - Meier survival analysis | | including patients with outcome prior to the time window |
|  | | Number of instances analysis | | including patients with outcome prior to the time window including patients with zero outcomes counts are grouped by date |
| All-Cause Hospitalization | | | | |
|  | **Outcome definition** | | | |
|  | | Visit | UMLS:HL7V3.0:VisitType:EMER | Visit: Emergency |
|  | | Visit | UMLS:HL7V3.0:VisitType:ACUTE | Visit: Inpatient Acute |
|  | | Visit | UMLS:HL7V3.0:VisitType:IMP | Visit: Inpatient Encounter |
|  | | Visit | UMLS:HL7V3.0:VisitType:NONAC | Visit: Inpatient Non-acute |
|  | | Visit | UMLS:HL7V3.0:VisitType:OBSENC | Visit: Observation Encounter |
|  | | Visit | UMLS:HL7V3.0:VisitType:SS | Visit: Short Stay |
|  | **Settings for the performed analyses** | | | |
|  | | Risk analysis | | including patients with outcome prior to the time window |
|  | | Kaplan - Meier survival analysis | | including patients with outcome prior to the time window |
|  | | Number of instances analysis | | including patients with outcome prior to the time window including patients with zero outcomes counts are grouped by date |
| Ventricular Arrhythmia | | | | |
|  | **Outcome definition** | | | |
|  | | Diagnosis | UMLS:ICD10CM:I47.0 | Re-entry ventricular arrhythmia |
|  | | Diagnosis | UMLS:ICD10CM:I47.2 | Ventricular tachycardia |
|  | | Diagnosis | UMLS:ICD10CM:I49.0 | Ventricular fibrillation and flutter |
|  | **Settings for the performed analyses** | | | |
|  | | Kaplan - Meier survival analysis | | including patients with outcome prior to the time window |
|  | | Number of instances analysis | | including patients with outcome prior to the time window including patients with zero outcomes counts are grouped by date |
|  | | Risk analysis | | including patients with outcome prior to the time window |
| Cardiac Arrest | | | | |
|  | **Outcome definition** | | | |
|  | | Diagnosis | UMLS:ICD10CM:I46.9 | Cardiac arrest, cause unspecified |
|  | **Settings for the performed analyses** | | | |
|  | | Kaplan - Meier survival analysis | | including patients with outcome prior to the time window |
|  | | Risk analysis | | including patients with outcome prior to the time window |
|  | | Number of instances analysis | | including patients with outcome prior to the time window including patients with zero outcomes counts are grouped by date |
| Syncope | | | | |
|  | **Outcome definition** | | | |
|  | | Diagnosis | UMLS:ICD10CM:R55 | Syncope and collapse |
|  | **Settings for the performed analyses** | | | |
|  | | Risk analysis | | including patients with outcome prior to the time window |
|  | | Number of instances analysis | | including patients with outcome prior to the time window including patients with zero outcomes counts are grouped by date |
|  | | Kaplan - Meier survival analysis | | including patients with outcome prior to the time window |

| **Outcome** | **Code** | **Description** |
| --- | --- | --- |
| AF Recurrence | ICD-10: I48.0, I48.1, I48.2, I48.91 | Diagnosis codes identifying recurrent atrial fibrillation |
| Cardioversion | CPT: 92960, 92961 | Electrical cardioversion procedures |

**Table S3: Baseline variables with their relevant codes**

| **Category** | **Code** | **Description** |
| --- | --- | --- |
| **Demographics** | AI | Age at Index |
|  | 2106-3 | White |
|  | 1002-5 | American Indian or Alaska Native |
|  | UNK | Unknown Race |
|  | F | Female |
|  | 2076-8 | Native Hawaiian or Other Pacific Islander |
|  | 2186-5 | Not Hispanic or Latino |
|  | 2135-2 | Hispanic or Latino |
|  | 2054-5 | Black or African American |
|  | M | Male |
|  | 2131-1 | Other Race |
|  | 2028-9 | Asian |
| **Diagnosis** | I10 | Essential (primary) hypertension |
|  | I50 | Heart failure |
|  | I49 | Other cardiac arrhythmias |
|  | I42 | Cardiomyopathy |
|  | E08-E13 | Diabetes mellitus |
|  | E65-E68 | Overweight, obesity and other hyperalimentation |
|  | I63 | Cerebral infarction |
|  | G45 | Transient cerebral ischemic attacks and related syndromes |
|  | I25 | Chronic ischemic heart disease |
|  | I70 | Atherosclerosis |
|  | I73 | Other peripheral vascular diseases |
|  | N17 | Acute kidney failure |
|  | N18 | Chronic kidney disease (CKD) |
|  | K70 | Alcoholic liver disease |
|  | K74.6 | Other and unspecified cirrhosis of liver |
|  | K72 | Hepatic failure, not elsewhere classified |
|  | K92.2 | Gastrointestinal hemorrhage, unspecified |
|  | I61 | Nontraumatic intracerebral hemorrhage |
|  | I60 | Nontraumatic subarachnoid hemorrhage |
|  | I21 | Acute myocardial infarction |
|  | I22 | Subsequent ST elevation (STEMI) and non-ST elevation (NSTEMI) myocardial infarction |
|  | E03.9 | Hypothyroidism, unspecified |
|  | E40-E46 | Malnutrition |
|  | J44 | Other chronic obstructive pulmonary disease |
|  | J45 | Asthma |
|  | G47.33 | Obstructive sleep apnea (adult) (pediatric) |
|  | I26-I28 | Pulmonary heart disease and diseases of pulmonary circulation |
|  | E78 | Disorders of lipoprotein metabolism and other lipidemias |
|  | D50 | Iron deficiency anemia |
|  | D60-D64 | Aplastic and other anemias and other bone marrow failure syndromes |
|  | D53 | Other nutritional anemias |
|  | D69.6 | Thrombocytopenia, unspecified |
|  | F10 | Alcohol related disorders |
|  | F17 | Nicotine dependence |
|  | F11 | Opioid related disorders |
|  | Z95.1 | Presence of aortocoronary bypass graft |
|  | Z95.8 | Presence of other cardiac and vascular implants and grafts |
|  | Z95.0 | Presence of cardiac pacemaker |
|  | Z99 | Dependence on enabling machines and devices, not elsewhere classified |
|  | Z51.5 | Encounter for palliative care |
| **Procedure** | 1013729 | Critical Care Services |
| **Medication** | CV100 | BETA BLOCKERS/RELATED |
|  | CV300 | ANTIARRHYTHMICS |
|  | CV800 | ACE INHIBITORS |
|  | CV250 | ANTIANGINALS |
|  | CV200 | CALCIUM CHANNEL BLOCKERS |
|  | CV805 | ANGIOTENSIN II INHIBITOR |
|  | CV700 | DIURETICS |
|  | BL110 | ANTICOAGULANTS |
|  | BL117 | PLATELET AGGREGATION INHIBITORS |
| **Laboratory** | 9028 | Potassium [Moles/volume] in Serum, Plasma or Blood |
|  | 9026 | Magnesium [Mass/volume] in Serum, Plasma or Blood |
|  | 9024 | Creatinine [Mass/volume] in Serum, Plasma or Blood |
|  | 9037 | Hemoglobin A1c/Hemoglobin.total in Blood |
|  | 9074 | Heart rate |
|  | 9083 | BMI |
|  | 9085 | Blood Pressure, Systolic |
|  | 9086 | Blood Pressure, Diastolic |
|  | 9005 | Troponin I.cardiac [Mass/volume] in Serum, Plasma or Blood |
|  | 9003 | Natriuretic peptide B [Mass/volume] in Serum, Plasma or Blood |
|  | 9032 | INR in Plasma or Blood |
|  | 9044 | Alanine aminotransferase [Enzymatic activity/volume] in Serum, Plasma or Blood |
|  | 9047 | Aspartate aminotransferase [Enzymatic activity/volume] in Serum or Plasma |
|  | 9050 | Bilirubin.total [Mass/volume] in Serum, Plasma or Blood |
|  | 9014 | Hemoglobin [Mass/volume] in Blood |
|  | 9020 | Platelets [#/volume] in Blood |

**Table S4: Detailed baseline characteristics of the study cohort before and after propensity score matching**

|  | **Before Propensity Matching** | | | | **After Propensity Matching** | | | |
| --- | --- | --- | --- | --- | --- | --- | --- | --- |
|  | **Early Rhythm Control**  **(n=341,560)** | **Early Rate Control**  **(n=504,214)** | **p-value** | **SMD** | **Early Rhythm Control**  **(n=200,631)** | **Early Rate Control**  **(n=200,631)** | **p-value** | **SMD** |
| **Demographics** | | | | | | | | |
| Age | 73.2 ± 8.9 | 75.8 ± 9.3 | <0.001 | 0.288 | 73.8 ± 9.1 | 74.0 ± 9.3 | <0.001 | 0.020 |
| White | 248,344 (72.7%) | 368,096 (73.0%) | 0.003 | 0.007 | 144,975 (72.3%) | 145,258 (72.4%) | 0.318 | 0.003 |
| American Indian or Alaska Native | 815 (0.2%) | 1,130 (0.2%) | 0.172 | 0.003 | 424 (0.2%) | 434 (0.2%) | 0.733 | 0.001 |
| Unknown Race | 39,227 (11.5%) | 61,677 (12.2%) | <0.001 | 0.023 | 25,574 (12.7%) | 25,470 (12.7%) | 0.622 | 0.002 |
| Female | 140,137 (41.0%) | 230,215 (45.7%) | <0.001 | 0.094 | 85,962 (42.8%) | 85,824 (42.8%) | 0.660 | 0.001 |
| Native Hawaiian or Other Pacific Islander | 1,876 (0.5%) | 3,045 (0.6%) | 0.001 | 0.007 | 1,051 (0.5%) | 1,062 (0.5%) | 0.810 | 0.001 |
| Not Hispanic or Latino | 238,543 (69.8%) | 343,203 (68.1%) | <0.001 | 0.038 | 137,263 (68.4%) | 138,057 (68.8%) | 0.007 | 0.009 |
| Hispanic or Latino | 11,259 (3.3%) | 15,525 (3.1%) | <0.001 | 0.012 | 6,226 (3.1%) | 5,959 (3.0%) | 0.014 | 0.008 |
| Black or African American | 26,893 (7.9%) | 37,521 (7.4%) | <0.001 | 0.016 | 14,332 (7.1%) | 14,397 (7.2%) | 0.691 | 0.001 |
| Male | 189,807 (55.6%) | 256,849 (50.9%) | <0.001 | 0.093 | 107,911 (53.8%) | 108,106 (53.9%) | 0.537 | 0.002 |
| Other Race | 8,312 (2.4%) | 12,871 (2.6%) | 0.001 | 0.008 | 5,071 (2.5%) | 5,140 (2.6%) | 0.489 | 0.002 |
| Asian | 16,093 (4.7%) | 19,874 (3.9%) | <0.001 | 0.038 | 9,204 (4.6%) | 8,870 (4.4%) | 0.011 | 0.008 |
| **Diagnosis** | | | | | | | | |
| Essential (primary) hypertension | 178,492 (52.3%) | 199,243 (39.5%) | <0.001 | 0.258 | 78,932 (39.3%) | 78,770 (39.3%) | 0.601 | 0.002 |
| Heart failure | 98,829 (28.9%) | 74,858 (14.8%) | <0.001 | 0.346 | 35,631 (17.8%) | 34,570 (17.2%) | <0.001 | 0.014 |
| Other cardiac arrhythmias | 68,287 (20.0%) | 52,517 (10.4%) | <0.001 | 0.269 | 25,621 (12.8%) | 24,768 (12.3%) | <0.001 | 0.013 |
| Cardiomyopathy | 29,494 (8.6%) | 14,891 (3.0%) | <0.001 | 0.245 | 8,497 (4.2%) | 8,260 (4.1%) | 0.061 | 0.006 |
| Diabetes mellitus | 90,860 (26.6%) | 90,710 (18.0%) | <0.001 | 0.208 | 37,935 (18.9%) | 37,577 (18.7%) | 0.148 | 0.005 |
| Overweight, obesity and other hyperalimentation | 59,067 (17.3%) | 49,353 (9.8%) | <0.001 | 0.221 | 22,247 (11.1%) | 21,766 (10.8%) | 0.015 | 0.008 |
| Cerebral infarction | 24,370 (7.1%) | 34,418 (6.8%) | <0.001 | 0.012 | 13,647 (6.8%) | 13,902 (6.9%) | 0.111 | 0.005 |
| Transient cerebral ischemic attacks and related syndromes | 9,847 (2.9%) | 11,365 (2.3%) | <0.001 | 0.040 | 4,623 (2.3%) | 4,609 (2.3%) | 0.883 | <0.001 |
| Chronic ischemic heart disease | 119,880 (35.1%) | 82,393 (16.3%) | <0.001 | 0.439 | 40,467 (20.2%) | 39,989 (19.9%) | 0.059 | 0.006 |
| Atherosclerosis | 28,366 (8.3%) | 19,339 (3.8%) | <0.001 | 0.188 | 9,105 (4.5%) | 8,996 (4.5%) | 0.407 | 0.003 |
| Other peripheral vascular diseases | 26,099 (7.6%) | 20,092 (4.0%) | <0.001 | 0.157 | 8,917 (4.4%) | 8,909 (4.4%) | 0.951 | <0.001 |
| Acute kidney failure | 82,326 (24.1%) | 56,944 (11.3%) | <0.001 | 0.340 | 30,182 (15.0%) | 28,719 (14.3%) | <0.001 | 0.021 |
| Chronic kidney disease (CKD) | 68,137 (19.9%) | 58,427 (11.6%) | <0.001 | 0.231 | 26,488 (13.2%) | 25,921 (12.9%) | 0.008 | 0.008 |
| Alcoholic liver disease | 2,855 (0.8%) | 2,854 (0.6%) | <0.001 | 0.032 | 1,303 (0.6%) | 1,243 (0.6%) | 0.233 | 0.004 |
| Other and unspecified cirrhosis of liver | 6,806 (2.0%) | 6,056 (1.2%) | <0.001 | 0.063 | 2,895 (1.4%) | 2,819 (1.4%) | 0.311 | 0.003 |
| Hepatic failure, not elsewhere classified | 9,889 (2.9%) | 3,858 (0.8%) | <0.001 | 0.159 | 3,020 (1.5%) | 2,751 (1.4%) | <0.001 | 0.011 |
| Gastrointestinal hemorrhage, unspecified | 13,965 (4.1%) | 12,129 (2.4%) | <0.001 | 0.095 | 5,539 (2.8%) | 5,421 (2.7%) | 0.253 | 0.004 |
| Nontraumatic intracerebral hemorrhage | 4,365 (1.3%) | 7,055 (1.4%) | <0.001 | 0.011 | 3,125 (1.6%) | 3,098 (1.5%) | 0.730 | 0.001 |
| Nontraumatic subarachnoid hemorrhage | 2,930 (0.9%) | 3,512 (0.7%) | <0.001 | 0.018 | 1,764 (0.9%) | 1,679 (0.8%) | 0.146 | 0.005 |
| Acute myocardial infarction | 49,109 (14.4%) | 24,145 (4.8%) | <0.001 | 0.330 | 13,397 (6.7%) | 13,282 (6.6%) | 0.466 | 0.002 |
| Subsequent ST elevation (STEMI) and non-ST elevation (NSTEMI) myocardial infarction | 520 (0.2%) | 137 (0.0%) | <0.001 | 0.042 | 88 (0.0%) | 99 (0.0%) | 0.421 | 0.003 |
| Hypothyroidism, unspecified | 40,172 (11.8%) | 45,684 (9.1%) | <0.001 | 0.089 | 19,040 (9.5%) | 18,608 (9.3%) | 0.019 | 0.007 |
| Malnutrition | 29,796 (8.7%) | 20,403 (4.0%) | <0.001 | 0.192 | 11,369 (5.7%) | 10,722 (5.3%) | <0.001 | 0.014 |
| Other chronic obstructive pulmonary disease | 52,791 (15.5%) | 50,291 (10.0%) | <0.001 | 0.165 | 22,044 (11.0%) | 21,444 (10.7%) | 0.002 | 0.010 |
| Asthma | 20,589 (6.0%) | 20,884 (4.1%) | <0.001 | 0.086 | 8,793 (4.4%) | 8,726 (4.3%) | 0.605 | 0.002 |
| Obstructive sleep apnea (adult) (pediatric) | 32,123 (9.4%) | 25,776 (5.1%) | <0.001 | 0.166 | 11,952 (6.0%) | 11,840 (5.9%) | 0.454 | 0.002 |
| Pulmonary heart disease and diseases of pulmonary circulation | 38,000 (11.1%) | 28,201 (5.6%) | <0.001 | 0.201 | 13,830 (6.9%) | 13,264 (6.6%) | <0.001 | 0.011 |
| Disorders of lipoprotein metabolism and other lipidemias | 154,424 (45.2%) | 154,025 (30.5%) | <0.001 | 0.306 | 64,210 (32.0%) | 63,425 (31.6%) | 0.008 | 0.008 |
| Iron deficiency anemia | 24,483 (7.2%) | 21,193 (4.2%) | <0.001 | 0.128 | 9,361 (4.7%) | 9,206 (4.6%) | 0.244 | 0.004 |
| Aplastic and other anemias and other bone marrow failure syndromes | 106,331 (31.1%) | 79,105 (15.7%) | <0.001 | 0.371 | 39,048 (19.5%) | 37,811 (18.8%) | <0.001 | 0.016 |
| Other nutritional anemias | 6,053 (1.8%) | 5,185 (1.0%) | <0.001 | 0.063 | 2,455 (1.2%) | 2,384 (1.2%) | 0.304 | 0.003 |
| Thrombocytopenia, unspecified | 34,254 (10.0%) | 18,726 (3.7%) | <0.001 | 0.252 | 10,737 (5.4%) | 10,246 (5.1%) | <0.001 | 0.011 |
| Alcohol related disorders | 12,950 (3.8%) | 13,566 (2.7%) | <0.001 | 0.062 | 5,727 (2.9%) | 5,498 (2.7%) | 0.028 | 0.007 |
| Nicotine dependence | 36,574 (10.7%) | 31,458 (6.2%) | <0.001 | 0.161 | 14,283 (7.1%) | 13,906 (6.9%) | 0.020 | 0.007 |
| Opioid related disorders | 2,783 (0.8%) | 2,437 (0.5%) | <0.001 | 0.041 | 1,081 (0.5%) | 1,107 (0.6%) | 0.577 | 0.002 |
| Presence of aortocoronary bypass graft | 25,104 (7.3%) | 12,931 (2.6%) | <0.001 | 0.222 | 6,555 (3.3%) | 6,572 (3.3%) | 0.880 | <0.001 |
| Presence of other cardiac and vascular implants and grafts | 15,874 (4.6%) | 8,422 (1.7%) | <0.001 | 0.171 | 5,118 (2.6%) | 4,849 (2.4%) | 0.006 | 0.009 |
| Presence of cardiac pacemaker | 10,303 (3.0%) | 13,552 (2.7%) | <0.001 | 0.020 | 5,581 (2.8%) | 5,574 (2.8%) | 0.946 | <0.001 |
| Dependence on enabling machines and devices, not elsewhere classified | 31,033 (9.1%) | 21,160 (4.2%) | <0.001 | 0.197 | 11,266 (5.6%) | 10,857 (5.4%) | 0.005 | 0.009 |
| Encounter for palliative care | 23,070 (6.8%) | 15,148 (3.0%) | <0.001 | 0.175 | 8,936 (4.5%) | 8,363 (4.2%) | <0.001 | 0.014 |
| **Procedure** | | | | | | | | |
| Critical Care Services | 66,047 (19.3%) | 41,153 (8.2%) | <0.001 | 0.329 | 22,978 (11.5%) | 22,292 (11.1%) | 0.001 | 0.011 |
| **Medications** | | | | | | | | |
| BETA BLOCKERS/RELATED | 175,428 (51.4%) | 40,143 (8.0%) | <0.001 | 1.080 | 36,966 (18.4%) | 38,136 (19.0%) | <0.001 | 0.015 |
| ANTIARRHYTHMICS | 138,620 (40.6%) | 108,735 (21.6%) | <0.001 | 0.420 | 52,154 (26.0%) | 51,244 (25.5%) | 0.001 | 0.010 |
| ACE INHIBITORS | 72,789 (21.3%) | 49,957 (9.9%) | <0.001 | 0.318 | 23,052 (11.5%) | 22,995 (11.5%) | 0.778 | 0.001 |
| ANTIANGINALS | 76,797 (22.5%) | 28,580 (5.7%) | <0.001 | 0.498 | 16,503 (8.2%) | 17,043 (8.5%) | 0.002 | 0.010 |
| CALCIUM CHANNEL BLOCKERS | 118,787 (34.8%) | 53,009 (10.5%) | <0.001 | 0.606 | 33,434 (16.7%) | 32,535 (16.2%) | <0.001 | 0.012 |
| ANGIOTENSIN II INHIBITOR | 55,102 (16.1%) | 34,837 (6.9%) | <0.001 | 0.292 | 17,227 (8.6%) | 17,018 (8.5%) | 0.238 | 0.004 |
| DIURETICS | 141,218 (41.3%) | 89,843 (17.8%) | <0.001 | 0.533 | 45,137 (22.5%) | 44,090 (22.0%) | <0.001 | 0.013 |
| ANTICOAGULANTS | 189,154 (55.4%) | 131,884 (26.2%) | <0.001 | 0.623 | 68,047 (33.9%) | 65,746 (32.8%) | <0.001 | 0.024 |
| PLATELET AGGREGATION INHIBITORS | 142,522 (41.7%) | 89,128 (17.7%) | <0.001 | 0.546 | 44,876 (22.4%) | 44,546 (22.2%) | 0.211 | 0.004 |
| **Laboratory** | | | | | | | | |
| Potassium [Moles/volume] in Serum, Plasma or Blood | 4.1 ± 0.6  240,369 (70.4%) | 4.1 ± 0.6  282,854 (56.1%) | <0.001 | 0.044 | 4.1 ± 0.6  113,229 (56.4%) | 4.1 ± 0.6  111,757 (55.7%) | <0.001 | 0.039 |
| 0 - 30 mmol/L | 240,369 (70.4%) | 282,854 (56.1%) | <0.001 | 0.299 | 113,229 (56.4%) | 111,757 (55.7%) | <0.001 | 0.015 |
| 30 - 60 mmol/L | 20 (0.0%) | 12 (0.0%) | 0.011 | 0.005 | 10 (0.0%) | 10 (0.0%) | 1 | <0.001 |
| 60 - 90 mmol/L | 10 (0.0%) | 10 (0.0%) | 0.381 | 0.002 | 0 (0.0%) | 10 (0.0%) | 0.002 | 0.010 |
| >90 mmol/L | 113 (0.0%) | 321 (0.1%) | <0.001 | 0.014 | 80 (0.0%) | 90 (0.0%) | 0.443 | 0.002 |
| Magnesium [Mass/volume] in Serum, Plasma or Blood | 2.0 ± 0.4  160,938 (47.1%) | 1.9 ± 0.4  121,275 (24.1%) | <0.001 | 0.171 | 2.0 ± 0.4  60,370 (30.1%) | 2.0 ± 0.4  57,872 (28.8%) | <0.001 | 0.037 |
| 0 - 1.70 mg/dL | 67,485 (19.8%) | 46,655 (9.3%) | <0.001 | 0.302 | 23,705 (11.8%) | 23,165 (11.5%) | 0.008 | 0.008 |
| 1.70 - 2.20 mg/dL | 138,670 (40.6%) | 98,730 (19.6%) | <0.001 | 0.471 | 49,476 (24.7%) | 48,086 (24.0%) | <0.001 | 0.016 |
| >2.20 mg/dL | 88,025 (25.8%) | 46,601 (9.2%) | <0.001 | 0.446 | 26,407 (13.2%) | 25,782 (12.9%) | 0.003 | 0.009 |
| Creatinine [Mass/volume] in Serum, Plasma or Blood | 1.4 ± 3.4  241,652 (70.7%) | 1.4 ± 5.6  283,248 (56.2%) | 0.681 | 0.001 | 1.4 ± 4.2  114,092 (56.9%) | 1.4 ± 5.0  112,468 (56.1%) | 0.227 | 0.005 |
| Hemoglobin A1c/Hemoglobin total in Blood | 6.4 ± 1.5  121,627 (35.6%) | 6.4 ± 1.6  101,912 (20.2%) | 0.006 | 0.012 | 6.4 ± 1.6  44,748 (22.3%) | 6.4 ± 1.6  44,847 (22.4%) | 0.062 | 0.012 |
| 4 - 5.60 % | 45,109 (13.2%) | 38,213 (7.6%) | <0.001 | 0.185 | 17,156 (8.6%) | 16,983 (8.5%) | 0.328 | 0.003 |
| 5.60 - 6.50 % | 66,318 (19.4%) | 54,607 (10.8%) | <0.001 | 0.241 | 23,933 (11.9%) | 23,841 (11.9%) | 0.654 | 0.001 |
| >6.50 % | 48,752 (14.3%) | 38,932 (7.7%) | <0.001 | 0.211 | 16,704 (8.3%) | 16,847 (8.4%) | 0.415 | 0.003 |
| Heart rate | 82.4 ± 22.4  162,619 (47.6%) | 79.8 ± 17.9  170,326 (33.8%) | <0.001 | 0.128 | 81.2 ± 21.8  72,066 (35.9%) | 80.0 ± 18.0  70,282 (35.0%) | <0.001 | 0.062 |
| BMI | 29.0 ± 7.1  182,620 (53.5%) | 28.8 ± 7.3  208,581 (41.4%) | <0.001 | 0.040 | 28.8 ± 7.1  85,672 (42.7%) | 28.8 ± 7.3  83,512 (41.6%) | 0.101 | 0.008 |
| 0 - 18.50 kg/m2 | 11,644 (3.4%) | 11,954 (2.4%) | <0.001 | 0.062 | 5,170 (2.6%) | 5,025 (2.5%) | 0.146 | 0.005 |
| 18.50 - 24.90 kg/m2 | 65,123 (19.1%) | 73,774 (14.6%) | <0.001 | 0.119 | 30,323 (15.1%) | 30,022 (15.0%) | 0.184 | 0.004 |
| 25 - 29.90 kg/m2 | 88,133 (25.8%) | 91,550 (18.2%) | <0.001 | 0.185 | 38,279 (19.1%) | 37,790 (18.8%) | 0.049 | 0.006 |
| 30 - 34.90 kg/m2 | 63,957 (18.7%) | 61,757 (12.2%) | <0.001 | 0.180 | 25,965 (12.9%) | 25,831 (12.9%) | 0.528 | 0.002 |
| 35 - 39.90 kg/m2 | 34,270 (10.0%) | 31,678 (6.3%) | <0.001 | 0.137 | 13,511 (6.7%) | 13,351 (6.7%) | 0.312 | 0.003 |
| >40 kg/m2 | 21,820 (6.4%) | 21,485 (4.3%) | <0.001 | 0.095 | 8,986 (4.5%) | 8,795 (4.4%) | 0.143 | 0.005 |
| Blood Pressure, Systolic | 120.6 ± 26.6  202,607 (59.3%) | 129.3 ± 23.1  241,140 (47.8%) | <0.001 | 0.350 | 123.3 ± 25.3  95,219 (47.5%) | 128.7 ± 23.6  93,777 (46.7%) | <0.001 | 0.222 |
| Blood Pressure, Diastolic | 66.6 ± 16.2  202,572 (59.3%) | 71.0 ± 14.3  241,216 (47.8%) | <0.001 | 0.284 | 68.0 ± 15.5  95,192 (47.4%) | 70.5 ± 14.6  93,780 (46.7%) | <0.001 | 0.169 |
| Troponin I cardiac [Mass/volume] in Serum, Plasma or Blood | 1.9 ± 12.4  65,882 (19.3%) | 0.8 ± 9.6  52,908 (10.5%) | <0.001 | 0.104 | 1.4 ± 11.0  24,780 (12.4%) | 1.0 ± 9.9  24,357 (12.1%) | <0.001 | 0.037 |
| Natriuretic peptide B [Mass/volume] in Serum, Plasma or Blood | 1012.2 ± 3430.1  68,218 (20.0%) | 814.2 ± 2814.4  54,609 (10.8%) | <0.001 | 0.063 | 871.1 ± 3131.6  24,955 (12.4%) | 903.5 ± 3084.2  24,692 (12.3%) | 0.246 | 0.010 |
| 0 - 100 pg/mL | 21,359 (6.3%) | 17,111 (3.4%) | <0.001 | 0.134 | 7,970 (4.0%) | 7,857 (3.9%) | 0.359 | 0.003 |
| 100 - 400 pg/mL | 30,674 (9.0%) | 24,086 (4.8%) | <0.001 | 0.167 | 10,985 (5.5%) | 10,822 (5.4%) | 0.256 | 0.004 |
| >400 pg/mL | 32,223 (9.4%) | 20,846 (4.1%) | <0.001 | 0.212 | 10,087 (5.0%) | 9,978 (5.0%) | 0.430 | 0.002 |
| INR in Plasma or Blood | 1.3 ± 0.6  192,834 (56.5%) | 1.4 ± 0.8  188,313 (37.3%) | <0.001 | 0.099 | 1.3 ± 0.7  82,304 (41.0%) | 1.3 ± 0.7  81,111 (40.4%) | 0.174 | 0.007 |
| 0.80 - 1.20 {INR} | 158,565 (46.4%) | 140,307 (27.8%) | <0.001 | 0.392 | 63,279 (31.5%) | 62,263 (31.0%) | 0.001 | 0.011 |
| 1.20 - 10 {INR} | 109,504 (32.1%) | 94,593 (18.8%) | <0.001 | 0.309 | 42,593 (21.2%) | 41,786 (20.8%) | 0.002 | 0.010 |
| Alanine aminotransferase [Enzymatic activity/volume] in Serum, Plasma or Blood | 47.7 ± 194.8  212,236 (62.1%) | 32.9 ± 108.9  234,893 (46.6%) | <0.001 | 0.094 | 44.2 ± 181.4  95,852 (47.8%) | 37.7 ± 143.1  94,199 (47.0%) | <0.001 | 0.040 |
| Aspartate aminotransferase [Enzymatic activity/volume] in Serum or Plasma | 61.1 ± 327.1  210,576 (61.7%) | 38.8 ± 144.8  233,216 (46.3%) | <0.001 | 0.088 | 57.5 ± 331.7  94,876 (47.3%) | 44.3 ± 184.8  93,279 (46.5%) | <0.001 | 0.049 |
| Bilirubin total [Mass/volume] in Serum, Plasma or Blood | 0.9 ± 2.0  206,225 (60.4%) | 0.9 ± 2.4  226,047 (44.8%) | <0.001 | 0.015 | 0.9 ± 2.3  92,242 (46.0%) | 0.9 ± 2.3  90,793 (45.3%) | 0.161 | 0.007 |
| Hemoglobin [Mass/volume] in Blood | 11.6 ± 2.6  239,918 (70.2%) | 12.3 ± 2.4  281,104 (55.8%) | <0.001 | 0.281 | 11.9 ± 2.5  113,004 (56.3%) | 12.1 ± 2.5  111,327 (55.5%) | <0.001 | 0.045 |
| Platelets [#/volume] in Blood | 210.5 ± 99.1  240,957 (70.5%) | 223.1 ± 93.3  282,544 (56.0%) | <0.001 | 0.131 | 218.0 ± 97.9  113,553 (56.6%) | 220.2 ± 96.7  111,859 (55.8%) | <0.001 | 0.023 |

*Abbreviation: SMD = standardized mean difference*

**Table S5: Comparison of outcomes after propensity score matching for subgroup Pharmacological Stratified**

| **Outcome** | **Atrial Fibrillation (AF)** | | **RD (95% CI)** | **RR (95% CI)** | **HR (95% CI)** | **p-value** |
| --- | --- | --- | --- | --- | --- | --- |
|  | **Early Rhythm Control**  **(n=181,591)** | **Early Rate Control**  **(n=181,591)** |  |  |  |  |
| All-Cause Mortality | 32,110 | 26,199 | 0.033  (0.030, 0.035) | 1.226  (1.207, 1.244) | 1.246  (1.226, 1.267) | <0.001 |
| Thromboembolism | 13,880 | 16,861 | −0.016  (−0.018, −0.015) | 0.823  (0.806, 0.841) | 0.820  (0.802, 0.838) | <0.001 |
| Major Bleeding | 12,415 | 13,197 | −0.004  (−0.006, −0.003) | 0.941  (0.919, 0.963) | 0.945  (0.922, 0.968) | <0.001 |
| All-Cause Hospitalizations | 78,299 | 76,567 | 0.010  (0.006, 0.013) | 1.023  (1.015, 1.030) | 1.034  (1.023, 1.044) | <0.001 |
| Cardiac Arrest | 4,904 | 2,138 | 0.015  (0.014, 0.016) | 2.294  (2.181, 2.412) | 2.324  (2.209, 2.445) | <0.001 |
| Ventricular Arrhythmia | 10,366 | 4,999 | 0.030  (0.028, 0.031) | 2.074  (2.006, 2.143) | 2.125  (2.055, 2.198) | <0.001 |
| Syncope | 7,877 | 7,296 | 0.003  (0.002, 0.005) | 1.080  (1.046, 1.114) | 1.093  (1.058, 1.128) | <0.001 |
| AF Recurrences* | 118,855 | 109,750 | 0.050  (0.047, 0.053) | 1.083  (1.078, 1.088) | 1.146  (1.136, 1.155) | <0.001 |
| Cardioversion* | 7,990 | 4,092 | 0.021  (0.020, 0.023) | 1.953  (1.881, 2.026) | 1.994  (1.920, 2.070) | <0.001 |

*Abbreviations:* *RD = risk difference; RR = risk ratio; HR = hazard ratio; CI = confidence interval*

**Follow-up period for these outcomes is 30 to 365 days*

**Table S6: Comparison of outcomes after propensity score matching for subgroup Procedure Stratified**

| **Outcome** | **Atrial Fibrillation (AF)** | | **RD (95% CI)** | **RR (95% CI)** | **HR (95% CI)** | **p-value** |
| --- | --- | --- | --- | --- | --- | --- |
|  | **Early Rhythm Control**  **(n=5,986)** | **Early Rate Control**  **(n=5,986)** |  |  |  |  |
| All-Cause Mortality | 94 | 473 | −0.063  (−0.071, −0.056) | 0.199  (0.160, 0.247) | 0.203  (0.163, 0.254) | <0.001 |
| Thromboembolism | 150 | 380 | −0.038  (−0.046, −0.031) | 0.395  (0.328, 0.475) | 0.398  (0.329, 0.480) | <0.001 |
| Major Bleeding | 77 | 276 | −0.033  (−0.039, −0.027) | 0.279  (0.217, 0.358) | 0.282  (0.219, 0.363) | <0.001 |
| All-Cause Hospitalizations | 1,508 | 2,315 | −0.135  (−0.151, −0.118) | 0.651  (0.617, 0.688) | 0.596  (0.559, 0.636) | <0.001 |
| Cardiac Arrest | 25 | 38 | −0.002  (−0.005, 0.000) | 0.658  (0.398, 1.088) | 0.677  (0.409, 1.122) | 0.128 |
| Ventricular Arrhythmia | 241 | 187 | 0.009  (0.002, 0.016) | 1.289  (1.068, 1.555) | 1.334  (1.102, 1.615) | 0.003 |
| Syncope | 216 | 239 | −0.004  (−0.011, 0.003) | 0.904  (0.755, 1.083) | 0.930  (0.774, 1.118) | 0.443 |
| AF Recurrences* | 4,180 | 3,600 | 0.097  (0.080, 0.114) | 1.161  (1.131, 1.192) | 1.373  (1.313, 1.436) | <0.001 |
| Cardioversion* | 457 | 184 | 0.046  (0.038, 0.054) | 2.484  (2.101, 2.936) | 2.631  (2.217, 3.122) | <0.001 |

*Abbreviations:* *RD = risk difference; RR = risk ratio; HR = hazard ratio; CI = confidence interval*

**Follow-up period for these outcomes is 30 to 365 days*

**Table S7: Comparison of outcomes after propensity score matching for subgroup 2015-2024 Stratified**

| **Outcome** | **Atrial Fibrillation (AF)** | | **RD (95% CI)** | **RR (95% CI)** | **HR (95% CI)** | **p-value** |
| --- | --- | --- | --- | --- | --- | --- |
|  | **Early Rhythm Control**  **(n=154,932)** | **Early Rate Control**  **(n=154,932)** |  |  |  |  |
| All-Cause Mortality | 27,893 | 22,467 | 0.035  (0.032, 0.038) | 1.242  (1.222, 1.262) | 1.269  (1.246, 1.291) | <0.001 |
| Thromboembolism | 11,985 | 14,981 | −0.019  (−0.021, −0.017) | 0.800  (0.782, 0.819) | 0.798  (0.779, 0.817) | <0.001 |
| Major Bleeding | 10,694 | 11,499 | −0.005  (−0.007, −0.003) | 0.930  (0.907, 0.954) | 0.937  (0.913, 0.962) | <0.001 |
| All-Cause Hospitalizations | 69,450 | 67,800 | 0.011  (0.007, 0.014) | 1.024  (1.016, 1.032) | 1.039  (1.028, 1.050) | <0.001 |
| Cardiac Arrest | 4,273 | 1,853 | 0.016  (0.015, 0.017) | 2.306  (2.185, 2.434) | 2.344  (2.220, 2.475) | <0.001 |
| Ventricular Arrhythmia | 8,980 | 4,295 | 0.030  (0.029, 0.032) | 2.091  (2.018, 2.167) | 2.151  (2.074, 2.231) | <0.001 |
| Syncope | 6,962 | 6,501 | 0.003  (0.002, 0.004) | 1.071  (1.036, 1.107) | 1.089  (1.052, 1.126) | <0.001 |
| AF Recurrences* | 104,858 | 97,517 | 0.047  (0.044, 0.051) | 1.075  (1.070, 1.081) | 1.148  (1.138, 1.158) | <0.001 |
| Cardioversion* | 7,480 | 3,741 | 0.024  (0.023, 0.025) | 1.999  (1.924, 2.078) | 2.053  (1.974, 2.135) | <0.001 |

*Abbreviations:* *RD = risk difference; RR = risk ratio; HR = hazard ratio; CI = confidence interval*

**Follow-up period for these outcomes is 30 to 365 days*

**Table S8: Comparison of outcomes after propensity score matching for subgroup Age 65-74 years Stratified**

| **Outcome** | **Atrial Fibrillation (AF)** | | **RD (95% CI)** | **RR (95% CI)** | **HR (95% CI)** | **p-value** |
| --- | --- | --- | --- | --- | --- | --- |
|  | **Early Rhythm Control**  **(n=52,223)** | **Early Rate Control**  **(n=52,223)** |  |  |  |  |
| All-Cause Mortality | 7,331 | 5,153 | 0.042  (0.038, 0.046) | 1.423  (1.376, 1.471) | 1.450  (1.399, 1.502) | <0.001 |
| Thromboembolism | 3,656 | 4,249 | -0.011  (-0.015, -0.008) | 0.860  (0.825, 0.898) | 0.860  (0.823, 0.899) | <0.001 |
| Major Bleeding | 3,028 | 3,177 | -0.003  (-0.006, 0.000) | 0.953  (0.908, 1.000) | 0.960  (0.913, 1.009) | 0.107 |
| All-Cause Hospitalizations | 22,969 | 22,125 | 0.016  (0.010, 0.022) | 1.038  (1.024, 1.053) | 1.061  (1.041, 1.081) | <0.001 |
| Cardiac Arrest | 1,742 | 692 | 0.020  (0.018, 0.022) | 2.517  (2.307, 2.747) | 2.558  (2.342, 2.793) | <0.001 |
| Ventricular Arrhythmia | 3,579 | 1,695 | 0.036  (0.033, 0.039) | 2.112  (1.996, 2.234) | 2.173  (2.051, 2.302) | <0.001 |
| Syncope | 1,877 | 1,815 | 0.001  (-0.001, 0.003) | 1.034  (0.971, 1.102) | 1.047  (0.982, 1.117) | 0.162 |
| AF Recurrences* | 36,079 | 32,920 | 0.060  (0.055, 0.066) | 1.096  (1.086, 1.106) | 1.180  (1.163, 1.198) | <0.001 |
| Cardioversion* | 3,063 | 1,955 | 0.021  (0.019, 0.024) | 1.567  (1.482, 1.656) | 1.600  (1.512, 1.693) | <0.001 |

*Abbreviations:* *RD = risk difference; RR = risk ratio; HR = hazard ratio; CI = confidence interval*

**Follow-up period for these outcomes is 30 to 365 days*

**Table S9: Comparison of outcomes after propensity score matching for subgroup Age 75-84 years Stratified**

| **Outcome** | **Atrial Fibrillation (AF)** | | **RD (95% CI)** | **RR (95% CI)** | **HR (95% CI)** | **p-value** |
| --- | --- | --- | --- | --- | --- | --- |
|  | **Early Rhythm Control**  **(n=67,146)** | **Early Rate Control**  **(n=67,146)** |  |  |  |  |
| All-Cause Mortality | 10,605 | 8,548 | 0.031  (0.027, 0.034) | 1.241  (1.208, 1.274) | 1.260  (1.225, 1.297) | <0.001 |
| Thromboembolism | 4,958 | 6,081 | -0.017  (-0.020, -0.014) | 0.815  (0.787, 0.845) | 0.811  (0.781, 0.842) | <0.001 |
| Major Bleeding | 4,498 | 4,805 | -0.005  (-0.007, -0.002) | 0.936  (0.900, 0.974) | 0.940  (0.902, 0.979) | 0.003 |
| All-Cause Hospitalizations | 29,083 | 28,449 | 0.009  (0.004, 0.015) | 1.022  (1.010, 1.035) | 1.032  (1.015, 1.049) | <0.001 |
| Cardiac Arrest | 1,790 | 775 | 0.015  (0.014, 0.017) | 2.310  (2.124, 2.511) | 2.339  (2.150, 2.545) | <0.001 |
| Ventricular Arrhythmia | 3,947 | 1,961 | 0.030  (0.027, 0.032) | 2.013  (1.909, 2.122) | 2.062  (1.953, 2.177) | <0.001 |
| Syncope | 2,820 | 2,589 | 0.003  (0.001, 0.006) | 1.089  (1.034, 1.148) | 1.102  (1.045, 1.162) | <0.001 |
| AF Recurrences* | 44,690 | 41,361 | 0.050  (0.044, 0.055) | 1.080  (1.072, 1.089) | 1.148  (1.133, 1.163) | <0.001 |
| Cardioversion* | 3,330 | 1,619 | 0.025  (0.023, 0.027) | 2.057  (1.940, 2.181) | 2.105  (1.984, 2.234) | <0.001 |

*Abbreviations:* *RD = risk difference; RR = risk ratio; HR = hazard ratio; CI = confidence interval*

**Follow-up period for these outcomes is 30 to 365 days*

**Table S10: Comparison of outcomes after propensity score matching for subgroup Age 85 years or above Stratified**

| **Outcome** | **Atrial Fibrillation (AF)** | | **RD (95% CI)** | **RR (95% CI)** | **HR (95% CI)** | **p-value** |
| --- | --- | --- | --- | --- | --- | --- |
|  | **Early Rhythm Control**  **(n=63,240)** | **Early Rate Control**  **(n=63,240)** |  |  |  |  |
| All-Cause Mortality | 13,759 | 12,141 | 0.026  (0.021, 0.030) | 1.133  (1.109, 1.158) | 1.144  (1.117, 1.172) | <0.001 |
| Thromboembolism | 5,116 | 6,424 | −0.021  (−0.024, −0.018) | 0.796  (0.769, 0.825) | 0.788  (0.760, 0.817) | <0.001 |
| Major Bleeding | 4,591 | 5,132 | −0.009  (−0.011, −0.006) | 0.895  (0.861, 0.929) | 0.893  (0.858, 0.929) | <0.001 |
| All-Cause Hospitalizations | 25,702 | 26,038 | −0.005  (−0.011, 0.000) | 0.987  (0.974, 1.000) | 0.977  (0.960, 0.994) | 0.008 |
| Cardiac Arrest | 1,246 | 602 | 0.010  (0.009, 0.012) | 2.070  (1.879, 2.280) | 2.084  (1.891, 2.297) | <0.001 |
| Ventricular Arrhythmia | 2,674 | 1,345 | 0.021  (0.019, 0.023) | 1.988  (1.864, 2.121) | 2.020  (1.891, 2.156) | <0.001 |
| Syncope | 3,077 | 2,829 | 0.004  (0.002, 0.006) | 1.088  (1.035, 1.143) | 1.094  (1.040, 1.151) | 0.001 |
| AF Recurrences* | 38,238 | 35,681 | 0.040  (0.035, 0.046) | 1.072  (1.062, 1.082) | 1.113  (1.097, 1.129) | <0.001 |
| Cardioversion* | 1,981 | 691 | 0.020  (0.019, 0.022) | 2.867  (2.631, 3.124) | 2.911  (2.670, 3.175) | <0.001 |

*Abbreviations:* *RD = risk difference; RR = risk ratio; HR = hazard ratio; CI = confidence interval*

**Follow-up period for these outcomes is 30 to 365 days*

**Figure S1: Kaplan-Meier Survival Analysis Plots for 1-Year Outcomes (Purple = Early Rhythm Control, Green = Early Rate Control)**


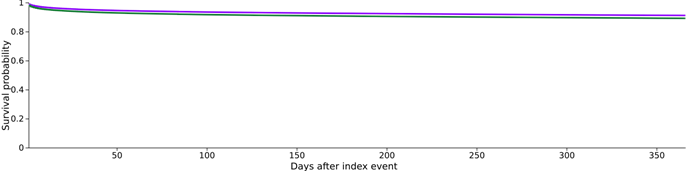


**Thromboembolism**


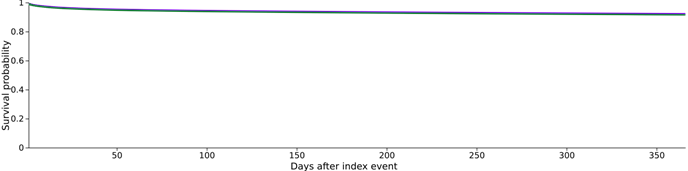


**Major bleeding**


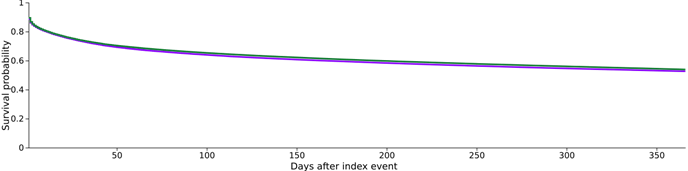


**All-cause hospitalization**


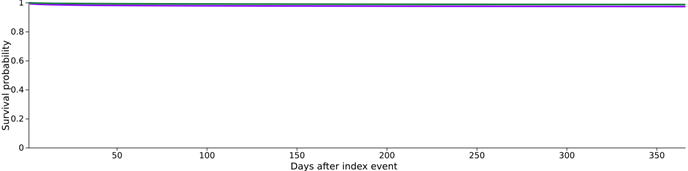


**Cardiac arrest**


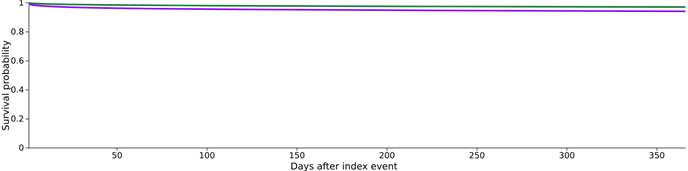


**Ventricular arrhythmia**


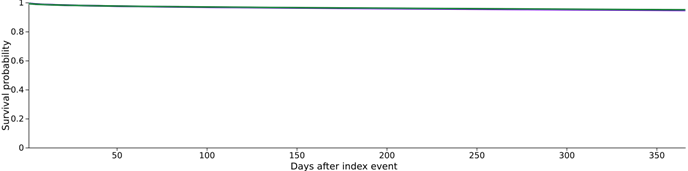


**Syncope**


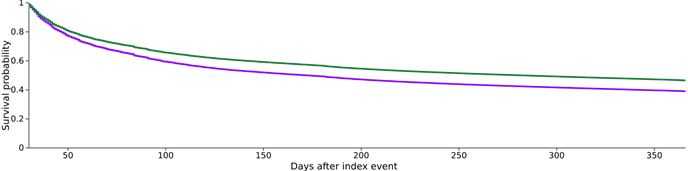


**AF recurrences**


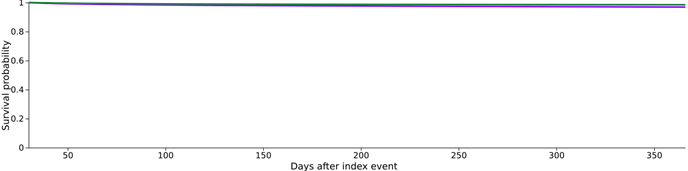


**Cardioversion**
